# Unanticipated Reactivity toward Nucleophilic Attack in the Synthesis of Saccharyl-1,3,4-Thiadiazolyl Conjugates: Structure and Mechanistic Insights

**DOI:** 10.1021/acs.joc.5c01116

**Published:** 2025-11-11

**Authors:** Bruno E. C. Guerreiro, Daniel F. Carvalho, Jaime A. S. Coelho, José A. Paixão, Luís M. T. Frija, Maria L. S. Cristiano

**Affiliations:** † Centro de Ciências do Mar (CCMAR), and Department of Chemistry and Pharmacy (FCT), 201875 University of Algarve, Campus de Gambelas, P-8005-039 Faro, Portugal; ‡ Institute of Molecular Sciences (IMS), Centro de Química Estrutural (CQE), Faculty of Sciences, 37809 University of Lisbon, Campo Grande, 1749-016 Lisboa, Portugal; § CFisUC, Department of Physics, 37829 University of Coimbra, 3004-516 Coimbra, Portugal; ∥ Institute of Molecular Sciences (IMS), Centro de Química Estrutural (CQE), Instituto Superior Técnico, 37809 University of Lisbon, 1049-001 Lisboa, Portugal

## Abstract

Along with the synthetic process optimization of 3-[(5-methyl-1,3,4-thiadiazole-2-yl)­sulfanyl]-1,2-benzothiazole 1,1-dioxide (MTSB), a selective copper chelator with potential interest in cancer chemotherapy, the unprecedented isolation of a novel compound, 3-(1,1-dioxidobenzo­[*d*]­isothiazol-3-yl)-5-methyl-1,3,4-thiadiazole-2­(3*H*)-thione (BMTT), evidenced an unexpected reactivity of the starting 5-methyl-1,3,4-thiadiazole-2-thiol. To shed light into the reaction mechanisms, quantum chemical calculations were conducted at the M06-2X/def2-TZVPP/PCM­(THF)//M06-2*X*/6-31++G­(d,p) level of theory. The results conjecture the formation of BMTT from nucleophilic attack of the nitrogen at position 3 of the thiadiazole ring, involved in an S-to-N delocalized thiadiazole-2-thiolate structure, which is thermodynamically more favorable in the presence of Na^+^. Experimental assays refute a plausible concerted 1,3-sigmatropic S- to N-rearrangement of MTSB that would lead to BMTT. Hence, contradicting the nucleophilicity indices of sulfur (from thiol) and nitrogen atoms of 5-methyl-1,3,4-thiadiazole-2-thiol, it is believed that an exotic nucleophilic attack by the nitrogen at 3-position of this reagent to the sp^2^ carbon in position 3 of pseudo-saccharyl chloride should take place. Besides, the crystal structures of the MTSB and BMTT hybrids were investigated in detail by X-ray crystallography.

## Graphical abstract

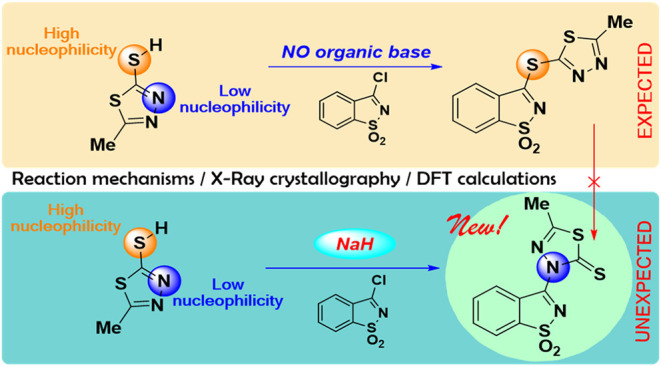

## Introduction

The design and synthesis of selective ligands for copper have attracted particular attention among medicinal chemists. Copper is a vital element in cellular metabolism,^1^ featuring in energy production cycles, iron homeostasis, and in the regulation of a variety of enzymes, as well as catalyzing antioxidants.^2–6^ Several works have reported an enhanced copper concentration in tumoral cells compared with normal cells, with Cu^2+^ being the main form present, given the hypoxic tumor microenvironment.^7–10^ Abnormal concentrations of copper ions were found to be closely correlated to carcinogenesis and tumor growth.^11^ Specifically, high concentrations of copper were found to balance the expression of the vascular endothelial growth factor (VEGF) and the hypoxia-inducible factor-1 (HIF-1), thus stimulating tumor angiogenesis.^12,13^

Given the impact of copper ions on cancer mechanisms, copper targeting emerged as an inspiring approach for anticancer therapy. Copper ionophores and copper chelators were designed to fight tumor progression by binding copper ions through different mechanisms. While copper chelators inhibit the copper-dependent cellular proliferation (cuproplasia) by scavenging copper, copper ionophores trigger the intracellular accumulation of copper, leading to cuproptosis, a unique type of cell death.^14–17^

Several copper chelators, quite diverse in their molecular structure, were reported for evidencing antitumor activity.^18–25^ However, many of these molecules also chelate with a variety of other metal ions, leading to substantial toxicity and side effects.^26–28^ Therefore, there is a growing need to develop safer and more selective copper chelators for use in cancer chemotherapy.

During the past decade, our laboratories were active on the design and synthesis of novel hybrid-azole-type ligands, in view of their possible application as chelators for cancer chemotherapy.^29^ Although the synthetic approach to most of the aforementioned molecules is quite straightforward, some processes for their synthesis have raised interesting challenges. Along with multistep synthesis efforts targeting intricate molecules, organic chemists may serendipitously discover unexpected structures that emerge from unknown reactivities. It may be challenging to clarify reaction mechanisms, but in addition to the chemists’ inherent curiosity about how the reactions occur, understanding the mechanisms is instrumental to expand the reactions’ potential. Computation has become a dominant tool to support mechanistic reasoning.^30^ As density functional theory (DFT) methods have become more able to deal with relatively large molecules, molecular orbital calculations emerged as an able partner with experiments in unraveling reaction mechanisms.

The hybrid-azole ligand 3-[(5-methyl-1,3,4-thiadiazol-2-yl)­sulfanyl]-1,2-benzothiazole 1,1-dioxide (MTSB; Scheme 1) was first synthesized and studied by some of us in 2018.^29c^ At that time, the synthesis of MTSB was achieved with an acceptable yield and without raising major difficulties. In vitro toxicity assays of MTSB showed copper concentration-dependent toxicity against cancer cells, without affecting normal cells. MTSB was shown to be most effective toward the hepatic (HepG2), neuroblastoma (SH-SY5), and lymphoma (U937) cell lines, thus representing a promising lead for copper-chelator-based cancer chemotherapy.

**Scheme 1:**
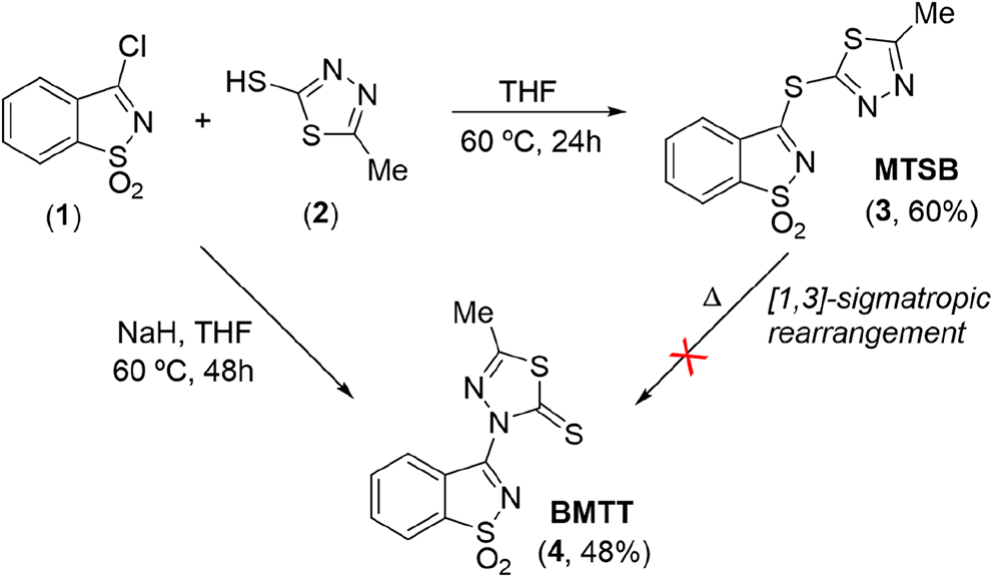
Illustrative Representation of the Synthetic Routes to MTSB and BMTT Molecules

With the aim of producing more MTSB for use in new toxicity tests, we envisaged improving the protocol initially applied in its synthesis. However, along the optimization of the synthetic method to access MTSB, a novel molecule was isolated, BMTT (Scheme 1), beyond the target compound. This article describes a new synthetic approach to MTSB, interrupted by an unanticipated and exotic nucleophilic attack by the nitrogen at position 3 of the thiadiazole ring to the carbon in position 3 of the saccharyl heterocycle, yielding BMTT. Theoretical calculations (DFT) were undertaken to shed light into the observed reactivity and to uncover a plausible mechanism. The structures of hybrid-azole ligands MTSB and BMTT were also studied by single-crystal X-ray crystallography.

## Results and Discussion

Primarily, the synthesis of the saccharyl-1,3,4-thiadiazole thioether MTSB (**3**; Scheme 1) proceeded without major difficulties, using dry THF as the solvent, at 60 °C, for 24 h. The reaction is triggered by a straightforward nucleophilic attack of the thiol substituent of thiadiazole (**2**) to the carbon in position 3 of the saccharyl chloride (**1**), leading to displacement of a chloride anion and genesis of the target compound, in reasonable yield.^29c^

Subsequent synthesis experiments aimed at improving the previous protocol resulted in the formation of a mixture of compounds that crystallized as yellow and red crystals. This observation prompted an optimization of the synthetic approach aiming to obtain, selectively, either yellow or red crystals.

In order to increase the nucleophilic character of the thiol’s sulfur atom, it was decided to use a base that would lead to the formation of the corresponding thiolate, expecting that the thiolate anion would promote the generation of MTSB at a higher yield. In this context, NaH and triethylamine were the first choices to test as bases.

Somewhat surprisingly, when triethylamine was used as a base, the target compound could not be isolated. Instead, formation of 3-diethylaminosaccharin^31^ was observed, resulting from the reaction between triethylamine and saccharyl chloride (**1**). This result led us to promptly discard the use of this organic base.

Also unexpected was the observed reactivity upon the use of NaH. When this strong base was used in the coupling reaction between pseudo-saccharyl chloride (**1**) and 5-methyl-1,3,4-thiadiazole-2-thiol (**2**), in THF, at 60 °C during 48 h, a new saccharin-1,3,4-thiadiazole conjugate (BMTT) was isolated (**4**, Scheme 1), resulting from nucleophilic attack of the nitrogen atom at position 3 of the thiadiazole ring (Scheme 1). We were not anticipating this result, given that the sulfur atom of the thiol function should present itself as a stronger nucleophile, compared to the nitrogen.

To the best of our knowledge, this is the first time that compound **4** has been reported. The molecule was isolated in a pure crystalline form, allowing structural characterization by single-crystal X-ray crystallography analysis, as described below.

### Single-Crystal X-ray Crystallography of MT SB and BMTT

For MTSB, a total of 182,772 reflections were measured up to θ = 27.985° (*R*_σ_ = 0.0075, *R*_int_ = 0.0430). The final quality factors of the refinement were *R*_1_(*I* > 2σ) = 0.0349, w*R*_all_ = 0.1046, and GOF = 1.064 for 5816 independent reflections and 165 refined parameters.

For BMTT, a total of 122,800 reflections were measured up to θ = 36.497° (*R*_σ_ = 0.0184, *R*_int_ = 0.0579). The final quality factors of the refinement were *R*_1_(*I* > 2σ) = 0.0305, w*R*_all_ = 0.0844, and GOF = 1.076 for 1536 independent reflections and 112 refined parameters.

Full details of the data collection and structure refinement procedures are provided as Supporting Information (Crystallographic Data). The CIF files containing the crystallographic data for the compounds BMTT and MTSB were deposited at the Cambridge Crystallographic Data Centre, with references 2416733 and 2416732, respectively. The performed single-crystal X-ray crystallography study unambiguously confirmed the conformations adopted by MTSB and BMTT in the crystalline state, as depicted by the ORTEP drawings shown in Figures 1 and 2. A summary of the single-crystal X-ray data collection and crystal structure refinement parameters is provided in Tables S1 and S2.

**Figure 1:**
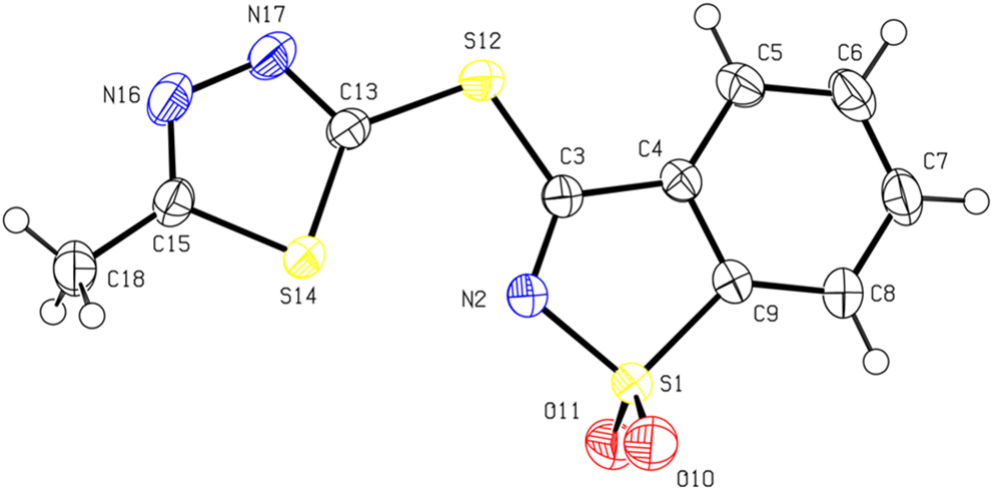
ORTEP drawing of the molecule of MTSB showing the atomic numbering scheme and the anisotropic displacement ellipsoids drawn at the 50% probability level.

**Figure 2:**
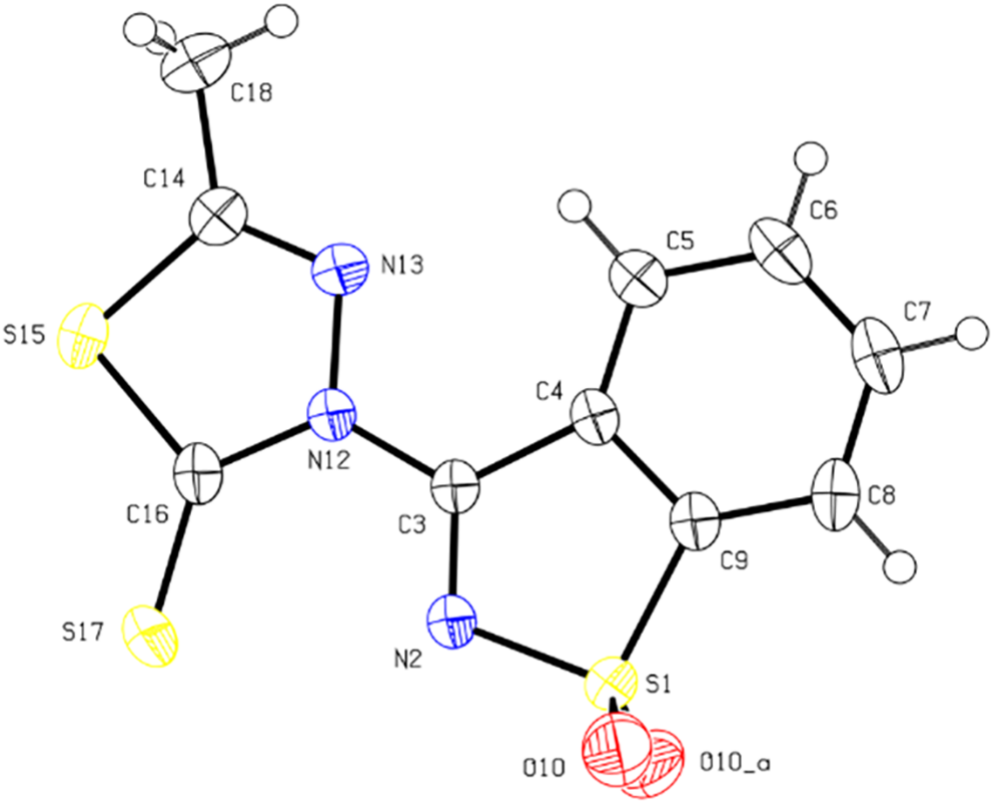
ORTEP drawing of the molecule of BMTT showing the atomic numbering scheme and the anisotropic displacement ellipsoids drawn at the 50% probability level.

BMTT crystallizes in the centric orthorhombic space group *Pnma*, with cell parameters *a* = 11.2516(19) Å, *b* = 6.7170(14) Å, *c* = 15.629(3) Å, and α = β = γ = 90°. The molecule lies on a crystallographic mirror plane. All H atoms could be located using the difference electron density maps. They were refined as riding on their parent atoms, with the exception to those of the methyl group, which had their coordinates freely refined, with one of the H atoms lying on the mirror plane and the two others as mirror images. Valence angles and bond lengths are unexceptional, S1–O10 [1.4219(15) Å] being significantly shorter than S1–N2 [1.6486(18) Å] and S–Csp^2^ bonds in the range [1.621(2)–1.745(2) Å], the shorter bond being S17–C16 and the longest being S1–C9. The N13–C14 [1.283(2) Å] and N2–C3 [1.290(3) Å] bonds are significantly shorter than a typical single Csp^2^–N bond, showing a significant double-bond character. The structure of BMTT contains no classical hydrogen bonds, and cohesion in the crystalline phase appears to be mainly dictated by van der Waals and π···π interactions. There is a short intramolecular interaction between the hydrogen atom attached to C5 and N13 [D···A: 2.877(3) Å]. Upon searching the structure for potential C–H···S hydrogen-bond interactions, only the short-contact C7–H7···S17­(*i*) [H8···S3:2.84 Å; C7···S17:3.774(2) Å, *i* = – 1 + *x*,*y*,*z* ] was found.

MTSB crystallizes in the centric monoclinic space group *P*2_1_/*c* with cell parameters *a* = 8.3564(14) Å, *b* = 9.4051(16) Å, *c* = 15.761(3) Å, α = 90°, β = 104.2980(10)°, and γ = 90°. All H atoms could be located on difference electron density maps. They were refined as riding on their parent atoms. The five- and six-membered rings of the fused benzothiazole group are coplanar, the angle between the least-squares planes of the two rings being 0.50(7)°. The least-squares planes of the five-membered thiadiazole ring and the fused nine-membered benzothiazole group are closely, but not strictly, coplanar, the dihedral angle being 11.07(7)°. The molecule has conformational flexibility for rotations around the C3–S12 and S12–C13 bonds, but the observed torsion angles S14–C13–S12–C3 [−1.20(14)°] and C13–S12–C3–N2 [12.36(12)°] show that the noncoplanarity arises mainly from rotation around the C3–S12 single bond. Similar to the BMTT, N16–C15 [1.2934(12) Å], N17–C13 [1.2973(17) Å], and N2–C3 [1.298(2) Å] are significantly shorter than a typical single Csp^2^–N bond, showing that the former three bonds have considerable double-bond character. Whereas in the BMTT, the two S–O bonds are equal by crystallographic symmetry, in the MTSB compound they are inequivalent, and a small asymmetry is observed between the S1–O10 and S1–O11 bond lengths [S1–O10:1.4266(13) Å; S1–O11:1.4362(13) Å]. The crystal structure features a short contact between one hydrogen atom of the methyl group and the N17 atom of a neighbor molecule that could be classified as a nonclassical hydrogen bond [H18B···N17:2.60 Å; C18···N17:3.538(2) Å (*i* = 2–*x*, 1/2 + *y*, 1/2–*z*)].

The structure of the MTSB is more compact and denser than that of the BMTT compound, but in both cases, there are no sizable voids in the structure that could accommodate solvent molecules.

Following the structural analysis of the molecules, and to understand which factors could be influencing the formation of the BMTT substrate to the detriment of MTSB, under certain reaction conditions, several experimental assays and theoretical DFT calculations were carried out.

### DFT Calculations

To gain insight into the role of the cation derived from the initially tested bases (Na^+^ or Et_3_NH^+^) in the formation of the thioether (MTSB) or the thione (BMTT), DFT calculations were performed at the M06-2X/def2-TZVPP/PCM­(THF)//M06-2*X*/6-31++G­(d,p) level of theory. The results point to nucleophilic coupling involving the sulfur or the nitrogen of the delocalized thiolate anion formed from the starting 5-methyl-1,3,4-thiadiazole-2-thiol. According to the calculations, a 1,3-heteroatom transposition of thiadiazole-2-thiol could occur, via a concerted mechanism proceeding through an activation barrier of 26.7 kcal mol^–1^ (see Figure S1). Regardless of the cation, the activation barrier for direct C–S bond formation (leading to MTSB) is 9–13 kcal mol^–1^ lower than that for direct C–N bond formation (leading to BMTT) (Figure 3, S2 and S3).

**Figure 3:**
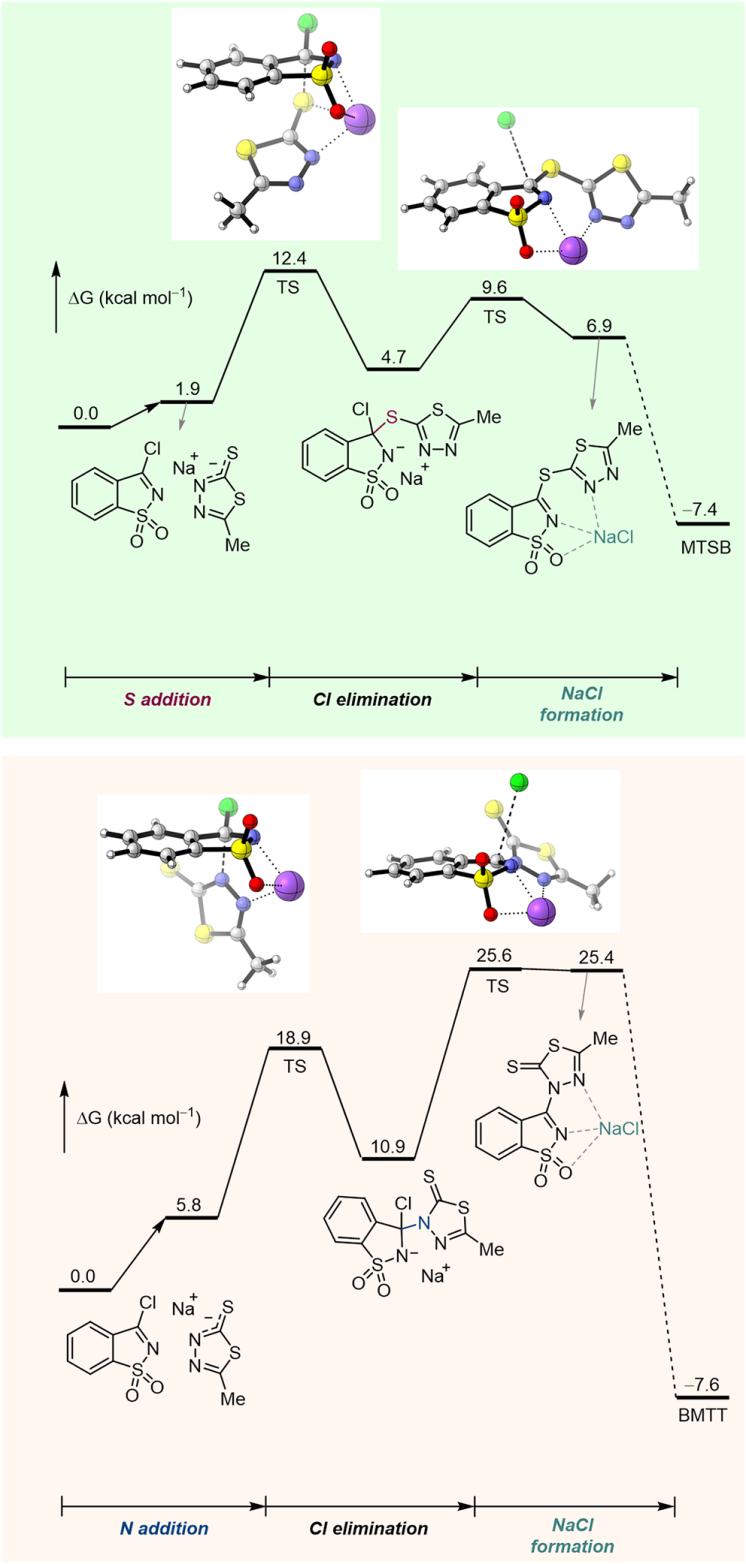
Gibbs free energy profiles for the formation of isomers MTSB and BMTT, in the presence of Na^+^. DFT calculations were performed at the M06-2X/def2-TZVPP/PCM­(THF)//M06-2*X*/6-31++G­(d,p) level of theory (energy values are given in kcal mol^–1^). [*For the complete Gibbs free energy profile, see* Figure S2].

This preference for sulfur addition aligns with the higher nucleophilicity calculated for the sulfur atom compared to nitrogen (Figure 4). These results also suggested that the formation of the C–N bond-linked conjugate (BMTT) could proceed via S–to–N isomerization, which is thermodynamically more favorable in the presence of Na^+^, despite the isomerization exhibiting similar activation barriers (25 to 27 kcal mol^–1^) for Na^+^ and [Et_3_NH]^+^.

**Figure 4:**
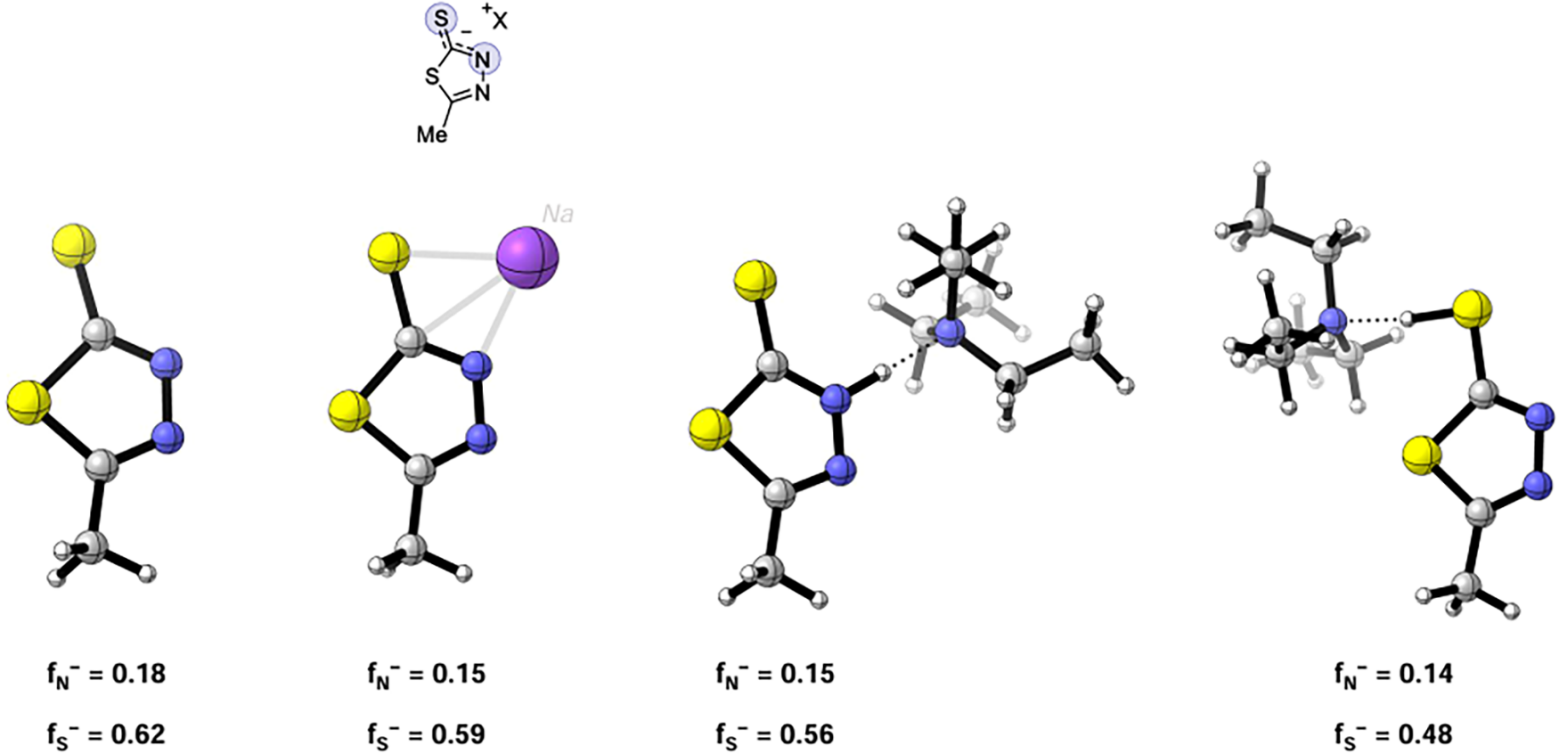
Fukui indices representing the nucleophilicity of sulfur and nitrogen atoms were determined at the M06-2X/def2-TZVPP//M06-2*X*/6-31++G­(d,p) level of theory.

Additionally, free energy calculations were performed for the thiadiazole substrate (**2**) coordinated to the Na^+^ ion, solvated by one, two, and three THF molecules (see Figure S4). Such calculations clearly show that adducts of **2** with explicit THF molecules are less favored than the isolated molecules since an increase in free energy is observed as the number of solvent molecules in the system increases. Besides, calculations carried out for the S addition step including the Na^+^ ion and THF explicit molecules revealed very low free energy variations (1–2 kcal mol^–1^) between the solvated and nonsolvated molecular systems (see Figure S5). Thus, analysis of the results extracted from the calculations including THF solvation leads us to conclude that the influence of the solvent in defining the reaction mechanism that operates will be residual.

### Mechanistic Considerations

DFT calculations assumed a possible concerted isomerization of the type 1,3-sigmatropic S- to N-rearrangement, with the consequent conversion of MTSB into BMTT. In this context, considering as hypothesis a thermally induced isomerization of thioether into thione, the initial substrate MTSB was heated under different conditions, as detailed in the Experimental and Computational Details Section (Experiments A, B and C). However, the results of the performed assays demonstrated that the isomerization of MTSB does not appear to take place since no signs of BMTT were detected. As such, we were unable to prove the theoretical hypothesis raised concerning the intervention of an isomerization mechanism.

To gather further knowledge on the potential reaction mechanism, we envisaged to evaluate the influence of the base used on the final reaction outcome. To this aim, we performed an assessment of the reaction conditions, testing a set of organic and inorganic bases in distinct solvents (see Table 1).

**Table 1:** Assessment of the Reaction Conditions^a^,^l^

entry	base (X equiv)	solvent	*T* (°C)	MTSB (%)^b^	BMTT (%)^b^
1	none	THF	60	60 ^29c^	n.d.
2^c^	NaH (1.0)	THF	60	n.d.	**48**
3^c^	Na_2_CO_3_ (1.0)	THF	60	n.d.	**43**
4^d^	K_2_CO_3_ (1.0)	THF	60	n.d.	n.d.
5^e^	LDA (1.0)	THF	60	n.d.	n.d.
6^f^	pyridine (1.2)	THF	60	**30**	d.n.i.
7^g^	pyridine (1.2)	THF	60	d.n.i.	d.n.i
8^h^	aniline (1.0)	THF	60	n.d.	n.d.
9	Et_3_N (1.0)	THF	60	n.d.	n.d.
10	NaH (1.0)	CH_3_CN	60	n.d.	**35**
11	NaH (1.0)	toluene	60	n.d.	**53**
12^i^	*none*	1,4-dioxane	80	**11**	n.d.
13^j^	pyridine (1.2)	1,4-dioxane	60	degrad.	n.d.
14^k^	pyridine + NaCl (1.2)	1,4-dioxane	60	degrad.	n.d.
15^k^	NaH (1.0)	1,4-dioxane	60	degrad.	n.d.

a Typical reaction conditions: thiadiazole-2 (4.51 mmol) was treated with a base (X equiv) in dry solvent (15.0 mL) under N2 atmosphere, at a specified temperature, for 1 h. A solution of saccharyl chloride (1) (4.96 mmol, 1.1 equiv) in dry solvent (15 mL) was then added to the initially prepared mixture. The reaction proceeded under stirring, at 60 °C, for 24 h.

b Isolated yield.

c Reaction time: 48 h.

d Isolation of a light brown powder NMR without signals corresponding to MTSB or BMTT.

e Isolation of a dark brown semiviscous paste (NMR without signals corresponding to MTSB or BMTT).

f Reaction time: 3 h. A mixture of BMTT and MTSB was obtained; MTSB was isolated in 30% yield.

g A mixture of BMTT and MTSB crystals was detected; unsuccessful product purification process.

h Formation of aniline-saccharinate, from reaction of the base with the saccharyl chloride substrate (see description in Experimental and Computational Details Section).

i Reaction time: 3 h.

j Isolation of yellow crystals. NMR indicating possible product degradation, but with some signals of MTSB.

k Isolation of yellow crystals. NMR spectra with deviation in the peak positions from the expected MTSB.

l n.d.: not detected; d.n.i.: detected and not isolated; degrad.: mixture degradation.

In a first assay, the use of Na_2_CO_3_ as a base in THF (Table 1, entry 3) led to the exclusive formation of the BMTT isomer in 43% yield, a result identical in every way to that initially obtained using NaH, under similar conditions (Table 1, entry 2). The use of K_2_CO_3_ and LDA as bases, which have different countercations than sodium, led to very impure reaction crudes, compromizing the isolation of the MTSB or BMTT isomers (Table 1, entries 4 and 5).

In addition to triethylamine, used in the first approach, the organic bases pyridine and aniline were then tested (Table 1, entries 6–8, 13, and 14). In contrast to what was observed with the use of triethylamine (i.e., the formation of 3-diethylaminosaccharin), the reaction using pyridine in THF, for 3 h, led to the formation of a mixture of MTSB and BMTT isomers with MTSB as the major compound, isolated in 30% yield (Table 1, entry 6). Surprisingly, extending the reaction time to 24 h, either in THF or 1,4-dioxane, led to a complex mixture, presumably due to the occurrence of unidentified cross reactions or product degradation (Table 1, entries 7 and 13). Likewise, when pyridine was used together with NaCl, in 1,4-dioxane, aiming to evaluate a potential influence of the sodium salt on reactivity, degradation of the reaction mixture was observed.

On the other hand, the use of aniline as base in THF led to a clean reaction, as was observed with triethylamine, but instead of the expected coupling between the thiadiazole and saccharin building blocks, a reaction between aniline and saccharyl chloride was observed, resulting in the formation of a new aniline-saccharinate (see NMR spectra in the Supporting Information).

Finally, the use of NaH as base was evaluated in different solvents, namely, toluene, acetonitrile, and 1,4-dioxane. Quite interestingly, also the use of toluene and acetonitrile as solvents led to the exclusive formation of the BMTT isomer (Table 1, entries 10 and 11), as observed for the reaction in THF. Once again, the reaction carried out in 1,4-dioxane, in the presence of a base, was not successful, revealing degradation of the reaction mixture.

Overall, the results of these experiments seem to clearly point to an influence of sodium ion on the thiadiazole activation process. Consequently, although the nucleophilic attack by the nitrogen atom at 3-position of the thiadiazole ring was not expected, given that the sulfur of the thiol has a much higher nucleophilic character, we propose that this mechanism should be at play, supported by the presence of Na^+^. Besides, we report that both the BMTS and BMTT molecules remained stable under normal solar radiation, ruling out a prospective light-mediated chemical transformation.

## Conclusions

In summary, a unique reaction was discovered by serendipity during the synthesis of the hybrid-azole derivative 3-[(5-methyl-1,3,4-thiadiazol-2-yl)­sulfanyl]-1,2-benzothiazole 1,1-dioxide (MTSB). The new molecule, dioxidobenzo­[*d*]­isothiazol-3-yl-5-methyl-1,3,4-thiadiazole-2­(3*H*)-thione (BMTT), should result from an unanticipated nucleophilic attack by the nitrogen at position 3 of 5-methyl-1,3,4-thiadiazole-2-thiol to pseudo-saccharyl chloride, promoted by Na^+^. Experimental evidence refuted a theoretically postulated isomerization mechanism via a concerted 1,3-sigmatropic S- to N-rearrangement, opened by DFT calculations.

The hybrid-azole compounds MTSB and BMTT could be isolated in a pure crystalline state, allowing a detailed structural study by using X-ray crystallography. In MTBS, the least-squares planes of the five-membered thiadiazole ring and the fused nine-membered benzothiazole groups are not coplanar, the dihedral angle being 11.07(7)°. The molecule has conformational flexibility for rotations around the C3–S12 and S12–C13 bonds forming the ether linkage (Figure 1) and the noncoplanarity arises mainly from rotation around the C3–S12 single bond. The crystal structure features a short contact between one hydrogen atom of the methyl group and the N17 atom of a neighbor molecule that could be classified as a nonclassical hydrogen bond. In BMTT, there is a short intramolecular interaction between the hydrogen atom attached to C5 and N13 (Figure 2). Cohesion in the crystalline phase of BMTT appears to be mainly dictated by van der Waals and π···π interactions. A search for C–H···S hydrogen-bond interactions revealed only the short-contact C7–H7···S17­(*i*). Although the structure of the MTSB is more compact and denser than that of the BMTT, there appear to be no sizable voids in the structures of both compounds that could accommodate solvent molecules.

Ultimately, the results of this investigation may have opened new insights into the synthesis of hybrid-azole derivatives containing the thiadiazole ring.

## Experimental and Computational Details

### Chemicals and Analytical Equipment

All reagents for the synthesis were purchased from commercial sources and used without further purification.

Analytical thin layer chromatography (TLC) was carried out using Merck (Darmstadt, Germany) TLC Silica gel 60 F_254_ aluminum sheets and visualized under UV or by appropriate stain (*p*-anisaldehyde and potassium permanganate were the most used). ^1^H and ^13^C nuclear magnetic resonance (NMR) spectra were recorded using a 400 MHz Bruker (Billerica, MA) instrument or a 500 MHz JEOL system (Peabody, MA) equipped with a Royal HFX probe, using DMSO-*D*_6_ as a deuterated solvent. All coupling constants are expressed in Hz and chemical shifts (δ) are described in parts per million (ppm), downfield from an internal standard of tetramethylsilane (TMS). Multiplicities are given as s (singlet), d (doublet), dd (double doublet), ddd (doublet, doublet, doublet) dt (double triplet), t (triplet), tt (triple triplet), and m (multiplet). Melting points (°C) were obtained on an SMP30 melting point apparatus and are uncorrected. High-resolution mass spectrometry (HRMS) was conducted on a Thermo Scientific High-Resolution Mass Spectrometer (HRMS) (Waltham, MA), model Orbitrap Elite, capable of MSn, *n* up to 10 (CCMar).

The single-crystal X-ray diffraction (XRD) study of compounds BMTT and MTSB was performed at room temperature. Data were collected on a Bruker APEX II diffractometer equipped with a 4K CCD detector, using graphite monochromated Mo Kα (λ = 0.71073 Å) radiation. Integration and correction for Lorentz and polarization factors were performed using SAINT V8.38A^32^ included in the APEXIII package.^33^ Absorption corrections, including odd and even spherical harmonics up to rank 3 and 6, respectively, were performed using SADABS-2016/2.^34^ The structure was solved by the dual-space algorithm implemented in SHELXT-2018/2,^35^ and the refinement of the structural model was performed using the full-matrix least-squares method employing SHELXL-2019/3.^35^ All nonhydrogen atoms were refined anisotropically. Hydrogen atoms were placed at calculated idealized positions and refined as riding using SHELXL-2019/3 default values, with the exception of those of the methyl group of BMTT, which had their coordinates freely refined, with one of the H atoms lying on the mirror plane and the two others as mirror images.

### Computational Details

Density functional theory (DFT) calculations were performed using the Gaussian 16 software package^36^ and structural representations were generated with *CYLview*.^37^ All the geometry optimizations were carried out using the hybrid meta-GGA functional M06-2X developed by Truhlar and co-workers^38,39^ and the valence double-ζ 6-31++G­(d,p) (6D, 7F) basis set. All the optimized geometries were verified by frequency computations as minima (zero imaginary frequencies) or transition states (a single imaginary frequency corresponding to the desired reaction coordinate). Single-point energy calculations on the optimized geometries were then evaluated using the same functional and the valence triple-ζ def2-TZVPP basis set,^40,41^ with solvent effects (tetrahydrofuran, THF) calculated by means of the polarizable continuum model (PCM) initially devised by Tomasi and co-workers.^42–45^ The free energy values presented in the manuscript and the Supporting Information were derived from the electronic energy values obtained at the M06-2X/def2-TZVPP/PCM­(THF) level and corrected by using the thermal and entropic corrections based on structural and vibration frequency data calculated at the M06-2*X*/6-31++G­(d,p) (6D, 7F) level.

### Synthesis of 3-Chloro-1,2-benzisothiazole 1,1-Dioxide (**1**)

We followed a previously reported procedure,^45^ with some modifications. Saccharin (20 g, 109 mmol) and phosphorus pentachloride (27.3 g, 131 mmol) were added to a round-bottom flask connected to a Graham condenser. The mixture was stirred at 220 °C for 4 h over a heating plate. Then, the reaction was allowed to cool to 180 °C and phosphorus oxychloride was distilled off from the reaction medium under vacuum. Once the reaction mixture was cooled to 100 °C, toluene (10 mL) was added. The final product crystallized upon cooling the mixture slowly to room temperature. The recovered crystals were washed with cold toluene (20 mL) and chloroform (30 mL), providing colorless needles (10.1 g; 50.1 mmol, 46% yield). Mp 143–145 °C. ^1^H NMR (500 MHz, DMSO-*D*_6_) δ 8.18 (d, *J* = 7.6 Hz, 1H), 8.04–7.98 (t, *J* = 8.0 Hz, 2H), 7.98–7.91 (m, 1H). ^13^C {^1^H} NMR (126 MHz, DMSO-*D*_6_) δ 160.7, 139.2, 135.8, 135.0, 127.4, 125.0, 121.3. Found: C, 41.5%; H, 2.0%; N, 6.9%; calcd for C_7_H_4_NO_2_SCl: C, 41.7%; H, 2.0%; N, 7.0%. MS (EI, *m*/*z*): 201 [M]^+^.

### Synthesis of 3-[(5-Methyl-1,3,4-thiadiazol-2-yl)­sulfanyl]-1,2-benzothiazole 1,1-Dioxide (**3**, MTSB)^29c^

#### Method A

A solution of 3-chloro-1,2-benzisothiazole 1,1-dioxide (**1**) (1.0 g, 4.97 mmol) and 5-methyl-1,3,4-thiadiazole-2-thiol (**2**) (0.65 g; 4.92 mmol) in dry THF (40 mL) was stirred at 60 °C under a nitrogen atmosphere. The reaction was monitored by TLC (ethyl acetate/hexane; 4:6). After 24 h, the solvent was evaporated under reduced pressure, and the remaining solid was dried under vacuum at room temperature. Crystallization from acetone/THF (2:1) yielded the required product as yellow crystals (1.0 g; 60% yield). Mp 212–214 °C. ^1^H NMR (500 MHz, DMSO-*D*_6_) δ 8.27 (d, *J* = 7.6 Hz, 1H), 8.16 (d, *J* = 7.6 Hz, 1H), 8.02–7.96 (m, 2H), 2.87 (s, 3H). ^13^C {^1^H} NMR (126 MHz, DMSO-*D*_6_) δ 173.2, 171.5, 153.1, 137.8, 135.7, 135.2, 128.9, 124.5, 122.8, 15.6. HRMS (ES^+^, *m*/*z*) calcd for C_10_H_8_N_3_O_2_S_3_ (M + H)^+^: 297.9779 u; found: 297.9775.

#### Method B

A solution of 3-chloro-1,2-benzisothiazole 1,1-dioxide (**1**) (1.00 g, 4.96 mmol) and 5-methyl-1,3,4-thiadiazole-2-thiol (**2**) (0.596 g, 4.51 mmol), in 1,4-dioxane (30 mL), was stirred at 80 °C, under a nitrogen atmosphere. The reaction was monitored by TLC (ethyl acetate/hexane; 4:6). After 3 h, cold water (30 mL) was added, resulting in the formation of a precipitate, which was filtered and dried under reduced pressure. The solid was recrystallized from acetone affording a mixture of yellow squared crystals, corresponding to the expected target compound (**3**, MTSB), and smaller red crystals that we were unable to separate. The physical separation of the larger yellow crystals provided 0.150 g (11% yield) of pure MTSB. Mp 212–214 °C. ^1^H NMR (500 MHz, DMSO-*D*_6_) δ 8.27 (d, *J* = 7.6 Hz, 1H), 8.16 (d, *J* = 7.6 Hz, 1H), 8.02–7.96 (m, 2H), 2.87 (s, 3H). ^13^C {^1^H} NMR (126 MHz, DMSO-*D*_6_) δ 173.2, 171.5, 153.1, 137.8, 135.7, 135.2, 128.9, 124.5, 122.8, 15.6. HRMS (ES^+^, *m*/*z*) calcd for C_10_H_8_N_3_O_2_S_3_ (M + H)^+^: 297.9779 u; found: 297.9775.

#### Method C

A mixture of 5-methyl-1,3,4-thiadiazole-2-thiol (0.596 g, 4.51 mmol) and pyridine (0.44 mL, 5.68 mmol), in dry THF (15 mL), was stirred at 60 °C for 30 min, under a nitrogen atmosphere. A solution of 3-chloro-1,2-benzisothiazole 1,1-dioxide (1.00 g, 4.96 mmol) in dry THF (15 mL) was then added. The reaction proceeded under stirring and monitored by TLC (ethyl acetate/hexane; 4:6), causing the formation of a precipitate. After 3 h, the precipitate was filtered, washed with cold water (30 mL), and dried under reduced pressure. The solid was recrystallized from acetone affording a mixture of yellow crystals and a few long and bright red needle-shaped crystals. Yellow crystals were then separated (0.41 g; 30% yield), corresponding to compound **3** (MTSB). Mp 208–211 °C. ^1^H NMR (500 MHz, DMSO-*D*_6_) δ 8.30–8.25 (m, 1H), 8.19–8.13 (m, 1H), 8.04–7.94 (m, 2H), 2.87 (s, 3H). ^13^C {^1^H} NMR (126 MHz, DMSO-*D*_6_) δ 173.1, 171.5, 153.1, 137.8, 135.6, 135.1, 128.9, 124.5, 122.8, 15.6.

### Procedure for the Formation of 3-(1,1-Dioxidobenzo­[*d*]­isothiazol-3-yl)-5-methyl-1,3,4-thiadiazole-2­(3*H*)-thione (4, BMTT) Using NaH as the Base

#### Method A

A mixture of 5-methyl-1,3,4-thiadiazole-2-thiol (0.596 g, 4.51 mmol) and NaH (60% w/w, 0.1804 g, 4.51 mmol) in dry THF (15 mL) was stirred at room temperature for 2 h under a nitrogen atmosphere. A solution of 3-chloro-1,2-benzisothiazole 1,1-dioxide (1.00 g, 4.96 mmol) in dry THF (15 mL) was then added. The final reaction mixture was stirred under reflux for 48 h. Cold water (30 mL) was added, resulting in the formation of a salmon color precipitate, which was filtered out and dried under reduced pressure. Recrystallization from acetone gave the pure target compound (**4**) as bright red needles (0.637 g, 48% yield). Mp 228–230 °C. ^1^H NMR (500 MHz, DMSO-*D*_6_) δ 8.51 (d, *J* = 7.4 Hz, 1H), 8.30 (d, *J* = 6.7 Hz, 1H), 7.95 (dd, *J* = 11.0, 7.5 Hz, 2H), 2.65 (s, 3H). ^13^C {^1^H} NMR (126 MHz, DMSO-*D*_6_) δ 189.4, 159.2, 157.7, 140.6, 134.7, 134.6, 130.4, 127.6, 122.8, 16.3. HRMS (ES^+^, *m*/*z*) calcd for C_10_H_8_N_3_O_2_S_3_ (M + H)^+^: 297.9779 u; found: 297.9778.

#### Method B

A mixture of 5-methyl-1,3,4-thiadiazole-2-thiol (0.596 g, 4.51 mmol) and NaH (60% w/w, 0.1804 g, 4.51 mmol) in dry acetonitrile (15 mL) was stirred at room temperature for 2 h under a nitrogen atmosphere. A solution of 3-chloro-1,2-benzisothiazole 1,1-dioxide (1.00 g, 4.96 mmol) in dry acetonitrile (15 mL) was then added. The final reaction mixture was stirred under reflux for 24 h. Cold water (30 mL) was added, resulting in the formation of a salmon color precipitate that was filtered out and dried under reduced pressure. Recrystallization from acetonitrile gave the pure target compound (**4**) as bright red needles (0.464 g, 35% yield). Mp 229–231 °C. ^1^H NMR (500 MHz, DMSO–D_6_) δ 8.50 (d, *J* = 9.0 Hz, 1H), 8.30 (d, *J* = 9.1 Hz, 1H), 7.96–7.91 (m, 2H), 2.65 (s, 3H). ^13^C {^1^H} NMR (126 MHz, DMSO-*D*_6_) δ 159.2, 134.5, 130.3, 122.8, 16.4.

#### Method C

A mixture of 5-methyl-1,3,4-thiadiazole-2-thiol (0.596 g, 4.51 mmol) and NaH (60% w/w, 0.1804 g, 4.51 mmol) in dry toluene (15 mL) was stirred at room temperature for 2 h under a nitrogen atmosphere. A solution of 3-chloro-1,2-benzisothiazole 1,1-dioxide (1.00 g, 4.96 mmol) in dry acetonitrile (15 mL) was then added. The final reaction mixture was stirred under reflux during 24 h. Cold water (30 mL) was added, resulting in the formation of a salmon color precipitate that was filtered out and dried under reduced pressure. Recrystallization from acetonitrile gave the pure target compound (**4**) as bright red needles (0.702 g, 53% yield). Mp 229–231 °C. ^1^H NMR (500 MHz, DMSO–D_6_) δ 8.50 (d, *J* = 7.8 Hz, 1H), 8.30 (d, *J* = 7.0 Hz, 1H), 7.96–7.91 (m, 2H), 2.65 (s, 3H). ^13^C {^1^H} NMR (126 MHz, DMSO-*D*_6_) δ 189.3, 157.7, 134.7, 134.6, 130.3, 129.7, 122.8, 16.3.

### Procedure for the Formation of 3-(1,1-Dioxidobenzo­[*d*]­isothiazol-3-yl)-5-methyl-1,3,4-thiadiazole-2­(3*H*)-thione (4, BMTT) Using Na_2_CO_3_ as the Base

A mixture of 5-methyl-1,3,4-thiadiazole-2-thiol (0.596 g, 4.51 mmol) and Na_2_CO_3_ (0.478 g, 4.51 mmol) in dry THF (15 mL) was stirred at 60 °C for 1 h under a nitrogen atmosphere. A solution of 3-chloro-1,2-benzisothiazole 1,1-dioxide (1.00 g, 4.96 mmol) in dry THF (15 mL) was then added. The reaction was monitored by TLC (ethyl acetate/hexane; 4:6) and proceeded under reflux with stirring during 48 h, causing the formation of a dark red precipitate. The precipitate was filtered, washed with cold water (30 mL), and dried under reduced pressure, allowing the isolation of a red powder (700 mg). A fraction of this powder (320 mg) was recrystallized from acetone and a second fraction (380 mg) from acetonitrile. Recrystallization from acetone gave rise to smaller, square crystals, while recrystallization from acetonitrile gave rise to long, thin needles, both red in color. After washing both crystals with ice-cold hexane and ice-cold acetone, 250 mg of the acetonitrile fraction and 280 mg of the acetone fraction were collected. Both isolated crystal forms were characterized as BMTT, representing a reaction yield of 43%. Mp 223–226 °C. ^1^H NMR (500 MHz, DMSO-*D*_6_) δ 8.53–8.47 (m, 1H), 8.33–8.27 (m, 1H), 7.95 (ddd, *J* = 11.4, 7.4, 1.3 Hz, 2H), 2.66 (s, 3H). ^13^C {^1^H} NMR (126 MHz, DMSO-*D*_6_) δ 189.1, 159.2, 157.6, 140.6, 134.7, 134.5, 130.3, 127.5, 122.8, 16.2.

### Procedures for the Investigation of a Potential Thermally Induced Isomerization of MTSB

#### Experiment A

A solution of pure thioether MTSB (20 mg, 6.73 × 10^–5^ mol) in dry THF (8 mL) was stirred at 60 °C under a nitrogen atmosphere. After 3 h, the reaction was stopped and the solvent dried under vacuum. Analysis of the resulting solid by NMR spectroscopy enabled the identification of the characteristic ^1^H NMR signals of the thioether MTSB at δ 8.27 (d, *J* = 7.6 Hz, 1H), 2.87 (s, 3H) and ^13^C {^1^H} NMR 15.6 while clearly showing the absence of any of the characteristic signals of the thione BMTT. The NMR spectra also showed some degradation of MTSB, possibly associated with cleavage of the thioether linkage to form saccharin. Recrystallization of the solid from acetone provided 14.5 mg of yellow crystals of the starting MTSB. Mp 206–209 °C.

#### Experiment B

A solution of the pure thioether MTSB (20 mg, 6.73 × 10^–5^ mol) and NaCl (4 mg, 6.84 × 10^–5^ mol) in dry THF (8 mL) was stirred at 60 °C under a nitrogen atmosphere. After 3 h, the reaction was stopped and filtered and the solvent dried under vacuum. Analysis of the resulting solid by NMR spectroscopy enabled the identification of the characteristic ^1^H NMR signals of the thioether MTSB at δ 8.27 (d, *J* = 7.6 Hz, 1H), 2.87 (s, 3H) and ^13^C {^1^H} NMR 15.6. No signs of the NMR signals characteristic of BMTT were observed. Recrystallization from acetone provided 17.5 mg of the starting MTBS as yellow crystals. Mp 206–209 °C.

#### Experiment C

A solution of the pure thioether MTSB (20 mg, 6.73 × 10^–5^ mol) and NaH (2.7 mg of 60% dispersion in mineral oil, 6.75 × 10^–5^ mol) in dry THF (8 mL) was stirred at 60 °C under a nitrogen atmosphere. After 3 h, the reaction was stopped and filtered. Cold water was added; no precipitate was formed, and the organic phase was extracted with ethyl acetate. The solvent was dried under vacuum, and a yellow oil was recovered. NMR spectra revealed product degradation, associated with cleavage of the thioether linkage to form saccharin. No signs of NMR signals characteristic of the thione BMTT were detected.

### Procedure for the Investigation of a Potential Thermally Induced Isomerization of BMTT

A solution of pure thione BMTT (40 mg, 1.35 × 10^–4^ mol) in 1,4-dioxane (10 mL) was stirred at 80 °C under a nitrogen atmosphere. After 24 h, the reaction was stopped and the solvent dried under vacuum. Analysis of the resulting solid by NMR spectroscopy enabled the identification of the characteristic ^1^H NMR signals of thione at δ 8.51 (d, 1H), 2.65 (s, 3H) and ^13^C {^1^H} NMR 16.3 while clearly showing the absence of any of the characteristic signals of the thioether MTSB. Recrystallization from acetone provided 12 mg of BMTT as red crystals. Mp 225–227 °C.

## Supplementary Material



## Data Availability

The data underlying this study are available in the published article and its Supporting Information.

## Notes

^1^H and ^13^C NMR, HRMS, and IR spectra of the synthesized compounds; tables with the summary of the single-crystal X-ray data collection and crystal structure refinement of **BMTT** and **MTSB**; Gibbs free energy profile for S to N isomerization; complete Gibbs free energy profiles for the formation of both isomers using [Et_3_NH]^+^ and Na^+^ ions; Gibbs free energy for adduct **2** and for the S addition step, including the Na^+^ ion with THF solvation; and associated Cartesian coordinates and energies (PDF)
