# Supplementary material for: Unanticipated Reactivity toward Nucleophilic Attack in the Synthesis of Saccharyl-1,3,4-Thiadiazolyl Conjugates: Structure and Mechanistic Insights
Source: J Org Chem. 2025 Nov 11;90(46):16301–9. doi: 10.1021/acs.joc.5c01116 (PMC12818759; doi:10.1021/acs.joc.5c01116)
Supplement: Supplementary file 1 [file jo5c01116_si_001.pdf]

Unanticipated Reactivity Towards Nucleophilic Attack in the Synthesis of Saccharyl-1,3,4-Thiadiazolyl Conjugates: Structure and Mechanistic Insights

Bruno E. C. Guerreiro[1], Daniel F. Carvalho[2], Jaime A. S. Coelho[2], José A. Paixão[3], Luís M. T. Frija*[4] and Maria L. S. Cristiano*[1]

[1] *Centro de Ciências do Mar (CCMAR), and Department of Chemistry and Pharmacy (FCT), University of Algarve, Campus de Gambelas, P-8005-039 Faro, Portugal*

[2] *Institute of Molecular Sciences (IMS), Centro de Química Estrutural (CQE), Faculty of Sciences, University of Lisbon, Campo Grande, 1749-016 Lisboa, Portugal*

[3] *CFisUC, Department of Physics, University of Coimbra, 3004-516 Coimbra, Portugal*

[4] *Institute of Molecular Sciences (IMS), Centro de Química Estrutural (CQE), Instituto Superior Técnico, University of Lisbon, 1049-001 Lisboa, Portugal*

Supporting information

Table of contents

HRMS spectra of the compounds **BMTT** and **MTSB**.	**S2**
^{1}H NMR and ^{13}C NMR spectra of compound **1**.	**S3**
^{1}H NMR and ^{13}C NMR spectra of the synthesized compounds.	**S4-S6**
Illustrative photos of MTSB and BMTT crystals.	**S7**
**Table S1.** Summary of the single-crystal X-ray data collection and crystal structure refinement of **BMTT**	**S8**
**Table S2.** Summary of the single-crystal X-ray data collection and crystal structure refinement of **MTSB**	**S9**
**Figure S1.** Gibbs free energy profile for S to N isomerization. DFT calculations were performed at the M06-2X/def2-TZVPP/PCM(THF)//M06-2X/6-31++G(d,p) or M06-2X/def2-TZVPP//M06-2X/6-31++G(d,p) (in brackets) level of theory (energy values in kcal mol^{-1}). Geometries depict the C–S and C–N bond distances in Å.	**S10**
**Figure S2.** Complete Gibbs free energy profile for the formation of both isomers using Na^{+}. DFT calculations were performed at the M06-2X/def2-TZVPP/PCM(THF)//M06-2X/6-31++G(d,p) level of theory (energy values in kcal mol^{-1}).	**S11**
**Figure S3.** Complete Gibbs free energy profile for the formation of both isomers using $[Et_3NH]^{+}$. DFT calculations were performed at the M06-2X/def2-TZVPP/PCM(THF)//M06-2X/6-31++G(d,p) level of theory (energy values in kcal mol^{-1}).	**S12**
**Figure S4.** Gibbs free energy of adduct **2** (thiadiazole coordinated to Na^{+}) solvated by one, two and three THF molecules.	**S13**
**Figure S5.** Gibbs free energy of S addition step with one and two THF explicit molecules.	**S14**
Cartesian coordinates and Energies for Figure S2.	**S15-S18**
Cartesian coordinates and Energies for Figure S3.	**S19-S24**

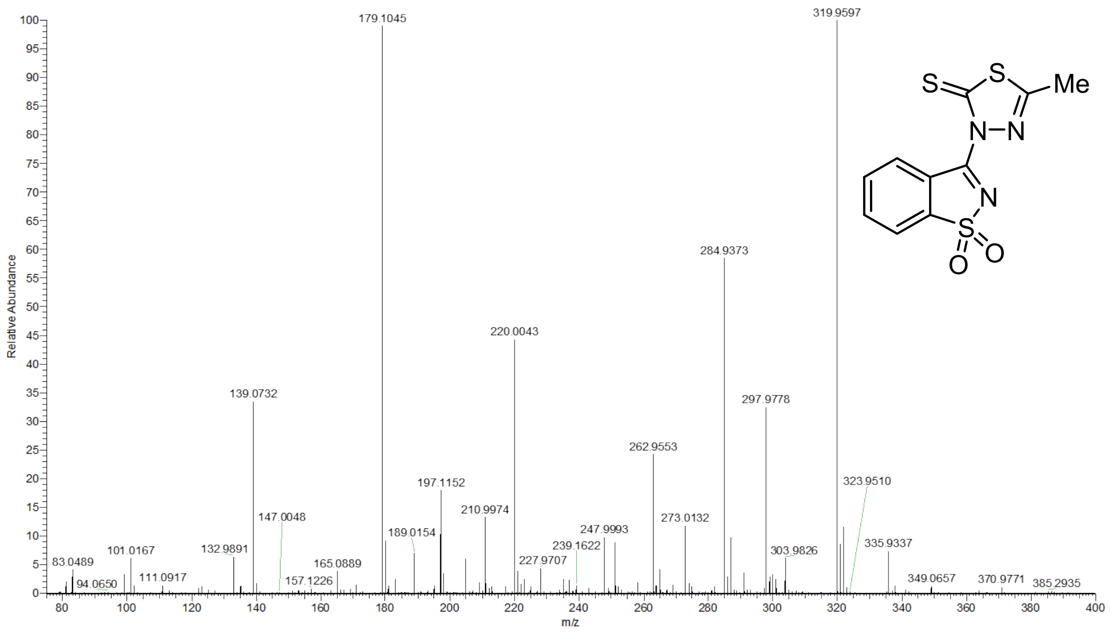

Electrospray ionization mass spectrum in positive-ion mode (HRMS-ES^{+}) of **BMTT** derivative.

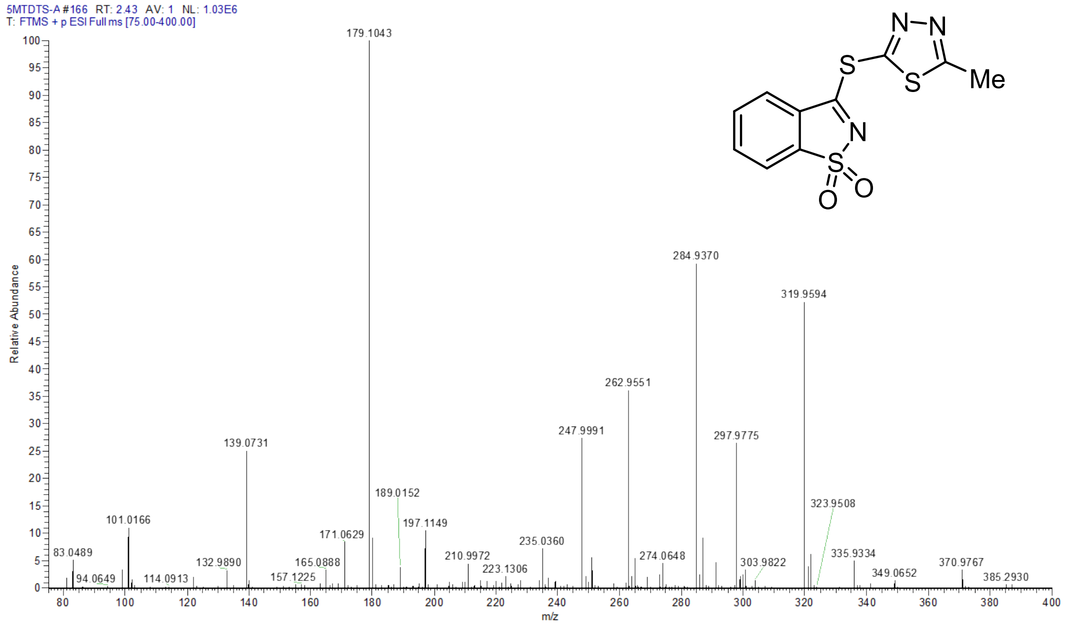

Electrospray ionization mass spectrum in positive-ion mode (HRMS-ES^{+}) of **MTSB** derivative.

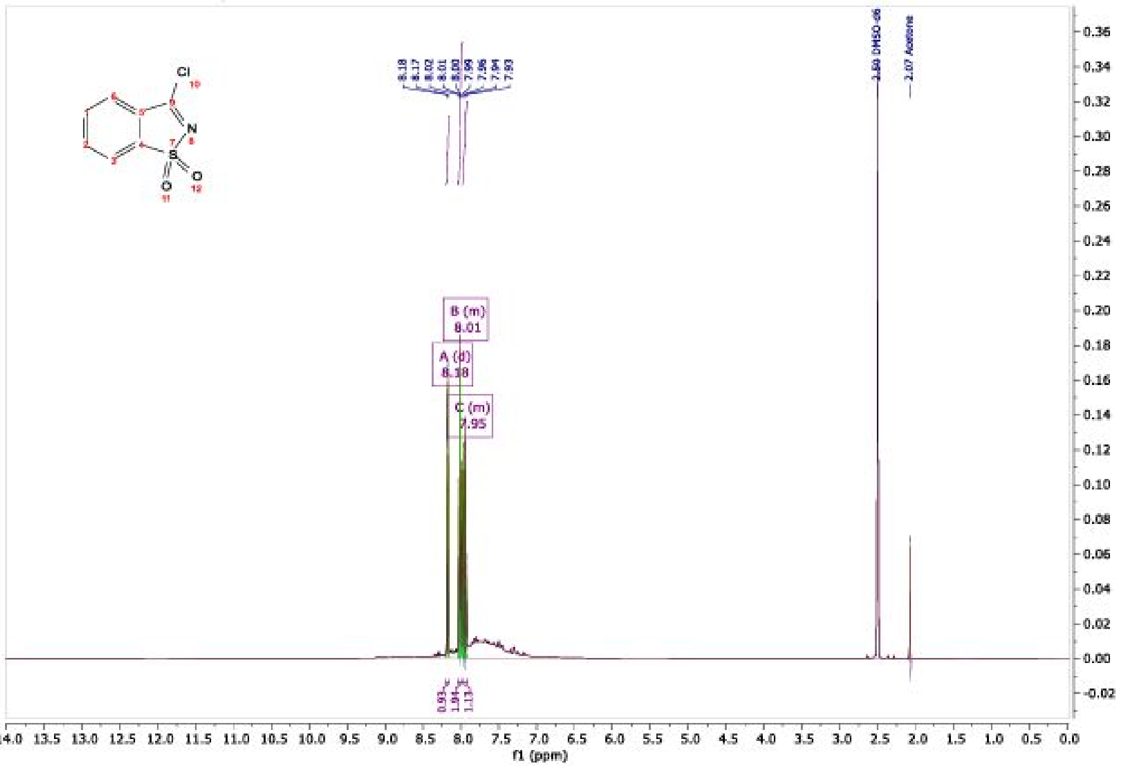

^{1}H NMR spectrum (500 MHz) of compound **1** in DMSO-d_6.

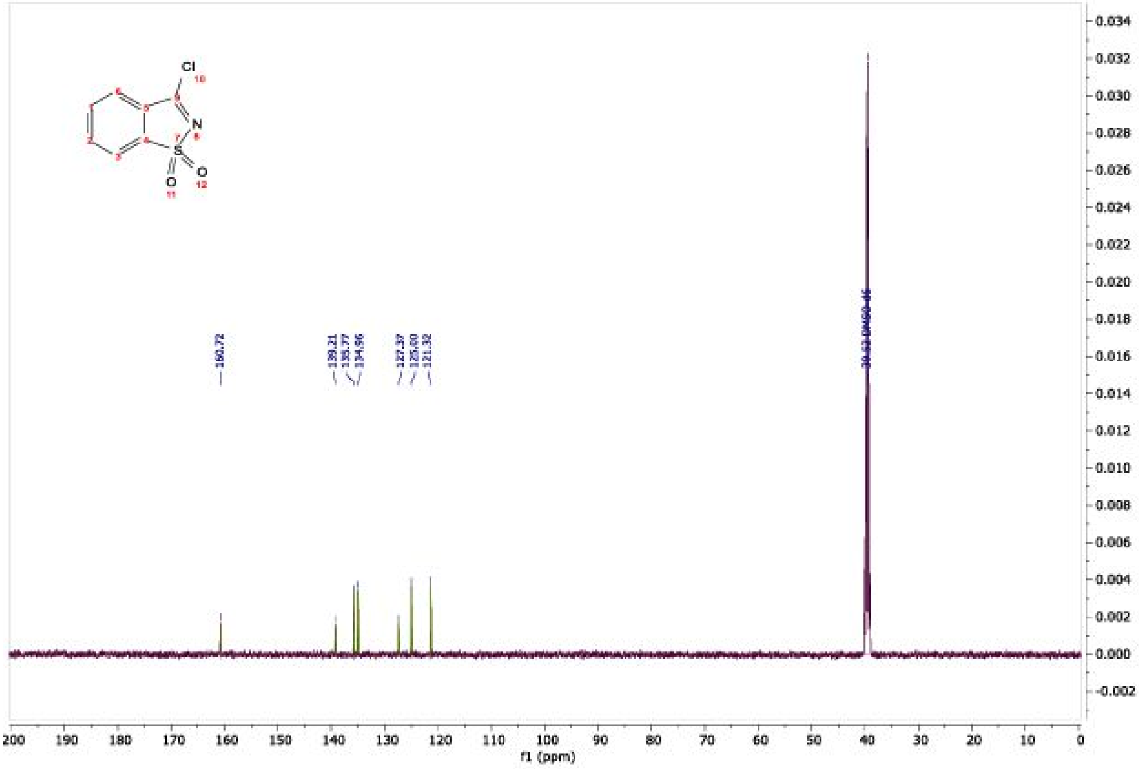

$^{13}C\{^{1}H\}$ NMR spectrum (126 MHz) of compound **1** in DMSO-d_6.

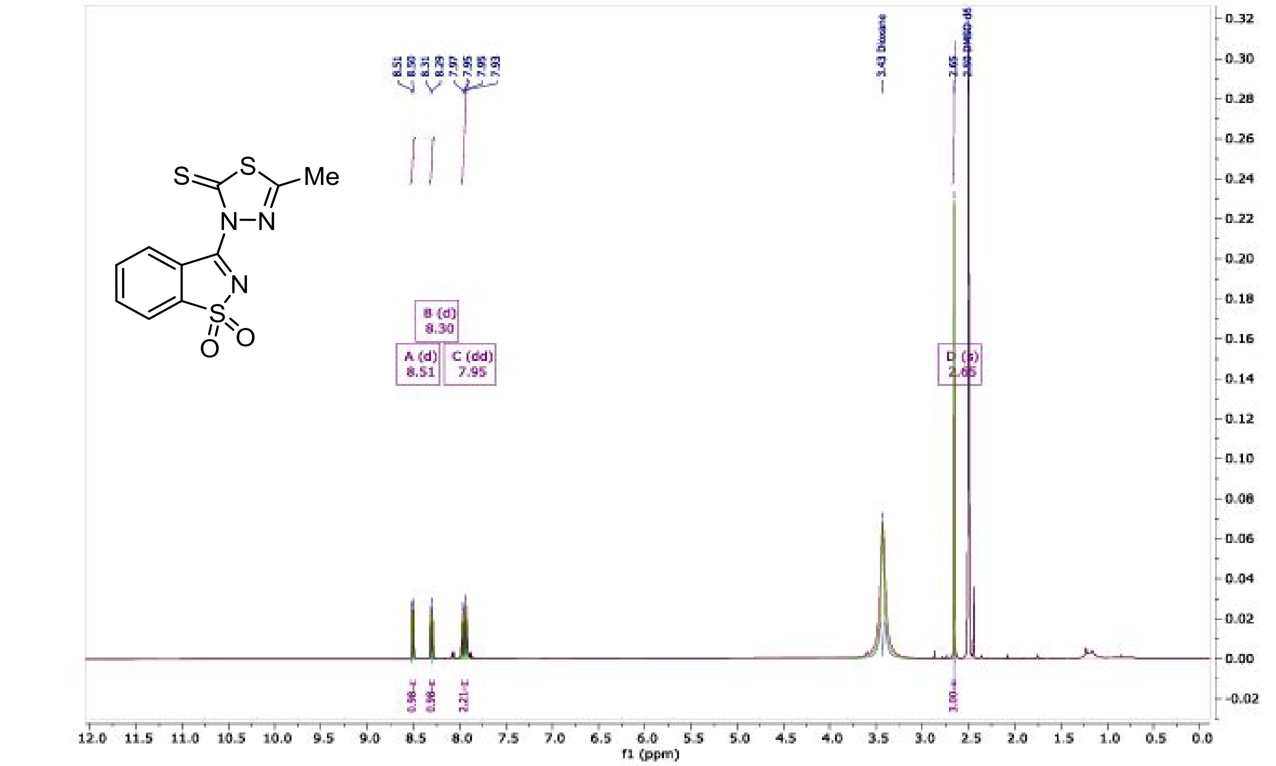

^{1}HNMR spectrum (500 MHz) of **BMTT** in DMSO-d_6.

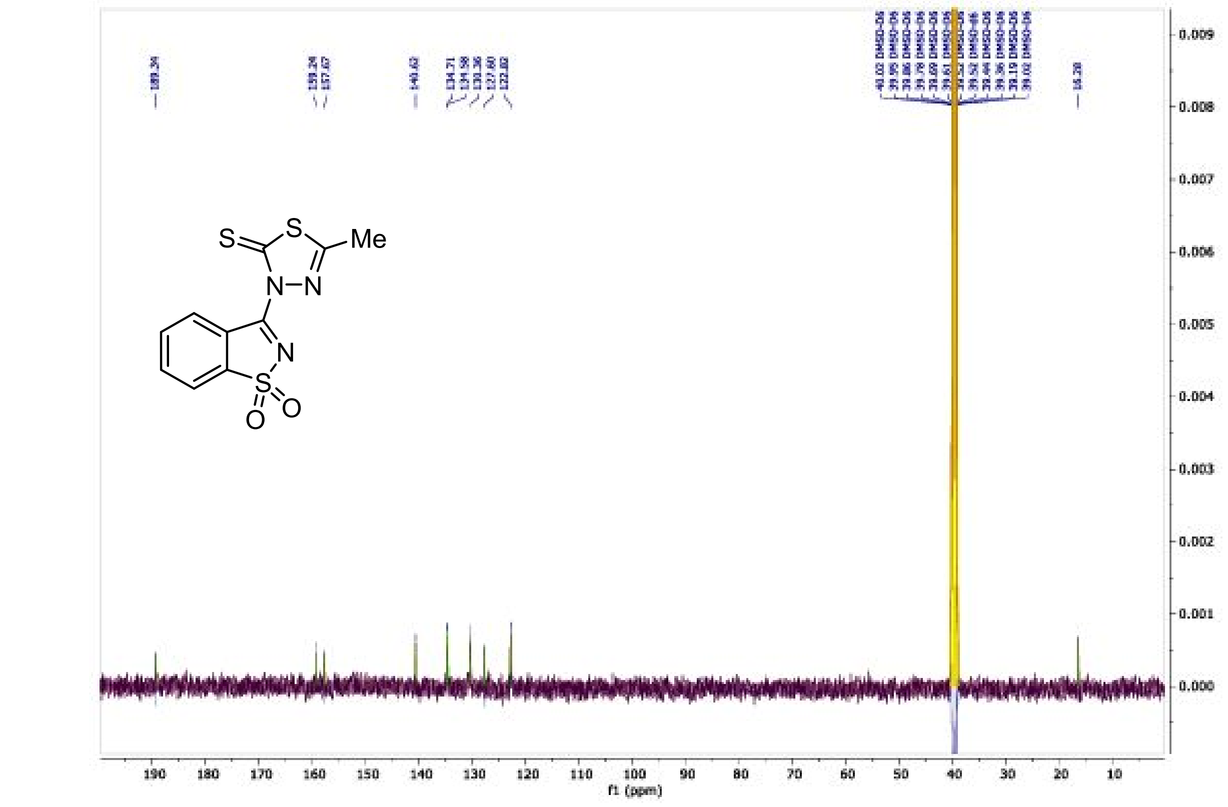

$^{13}C\{^1H\}$ NMR spectrum (126 MHz) of **BMTT** in DMSO-d_6.

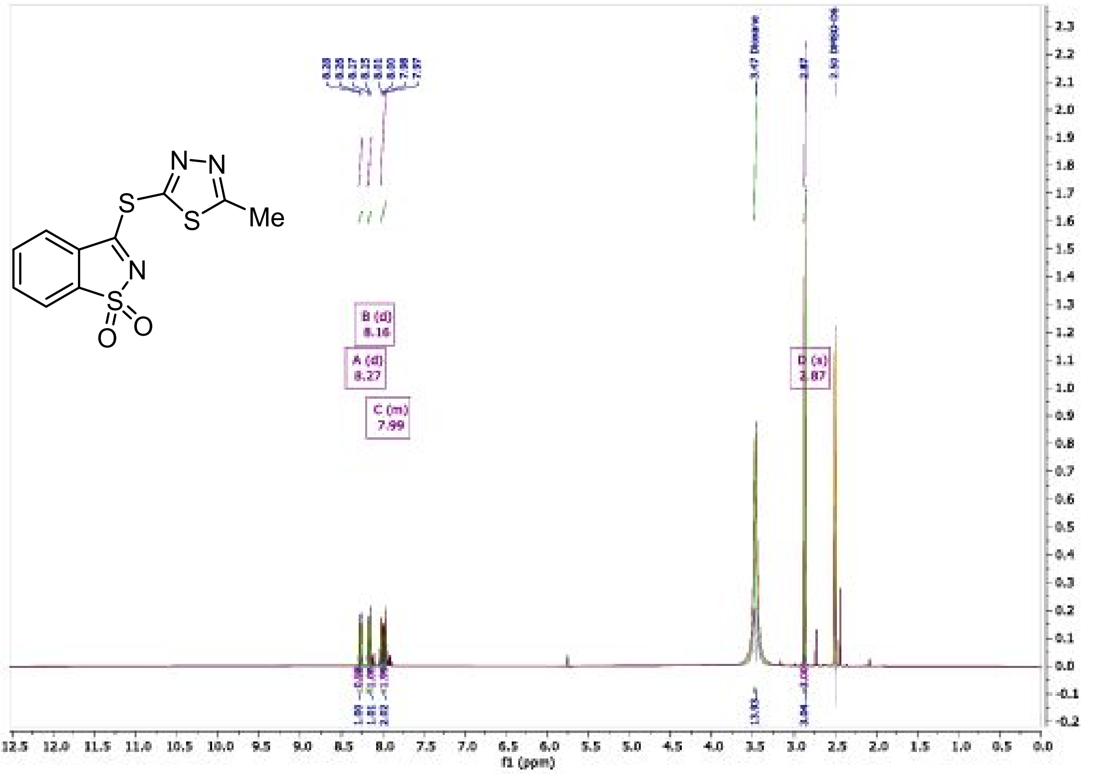

^{1}H NMR spectrum (500 MHz) of **MTSB** in DMSO-d_6.

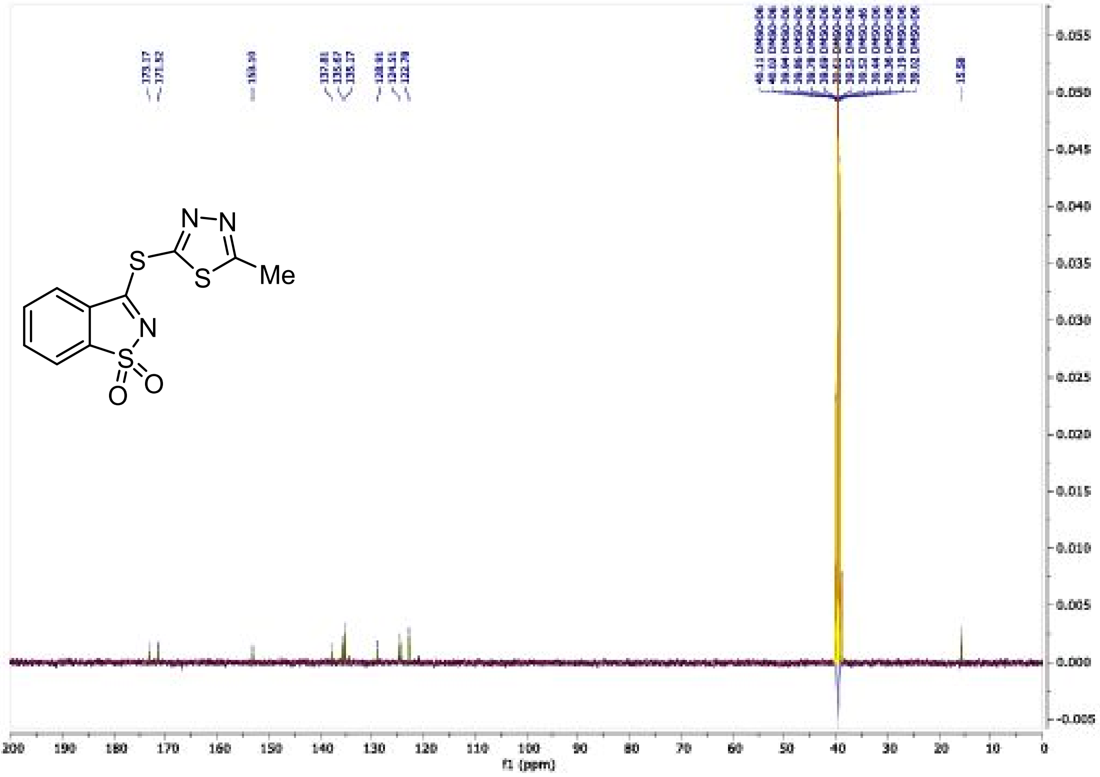

^{13}C{^{1}H} NMR spectrum (126 MHz) of **MTSB** in DMSO-d_6.

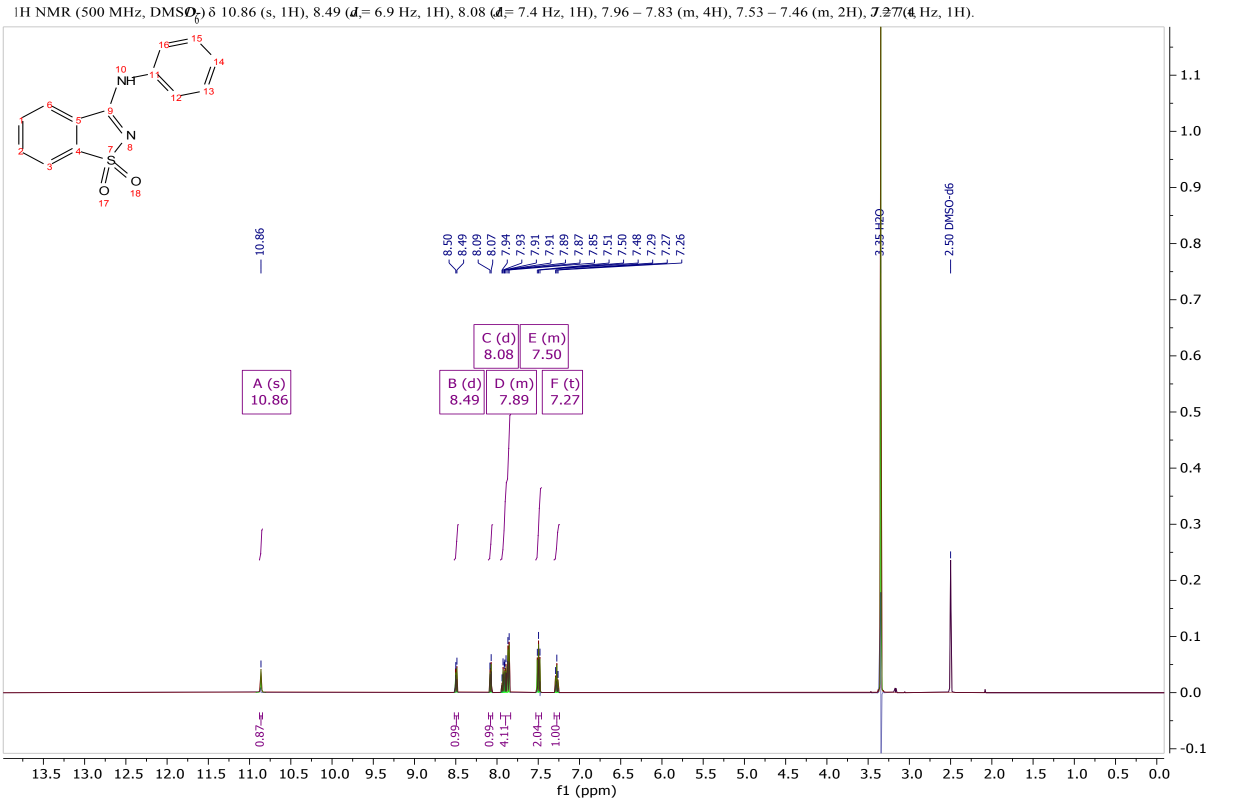

^{1}H NMR spectrum (500 MHz) of **saccharinate-aniline** derivative in DMSO-d_6.
[*Compound isolated when aniline was used as base*]

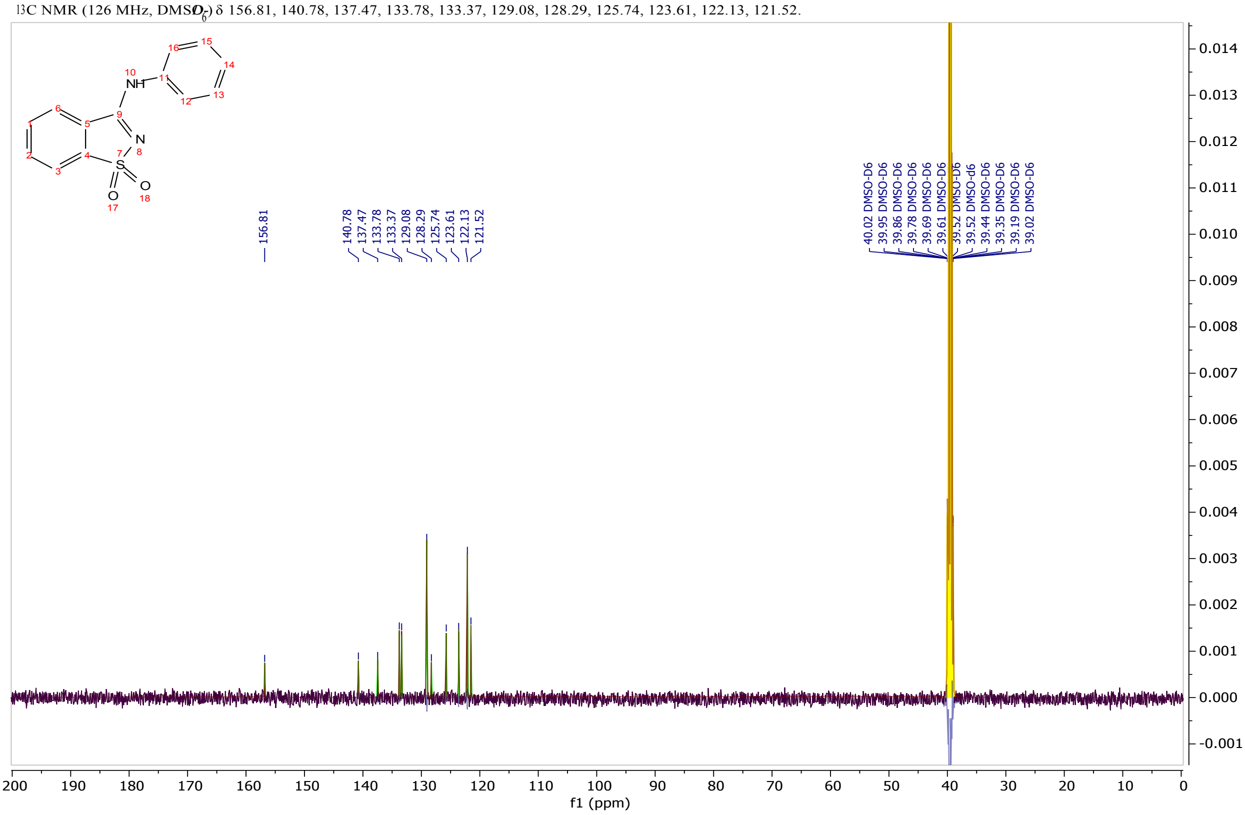

^{13}C{^{1}H} NMR spectrum (126 MHz) of **saccharinate-aniline** derivative in DMSO-d_6.

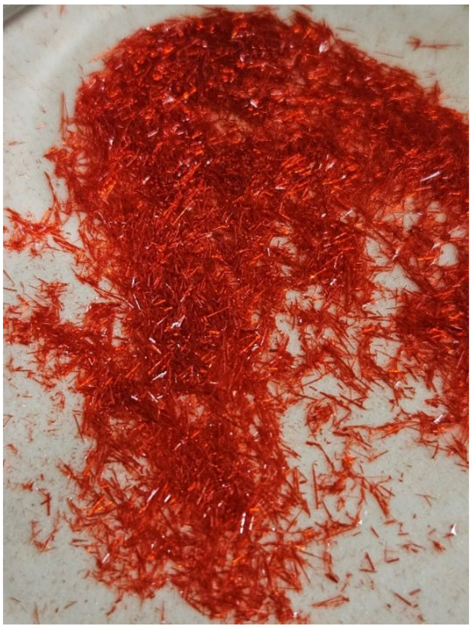 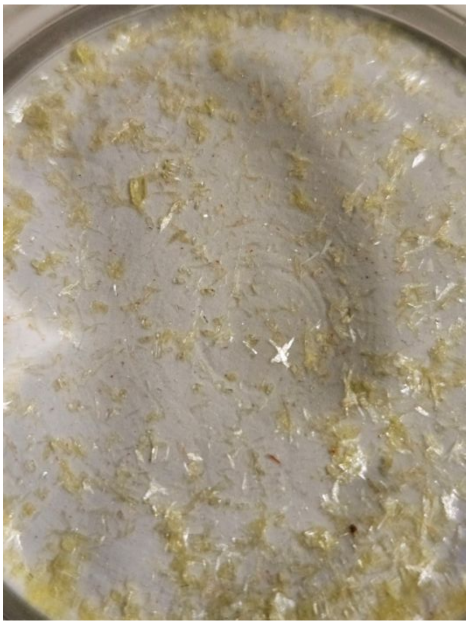

Left: BMTT red crystals (Synthesis in THF using NaH or Na_2CO_3);

Right: MTSB yellow crystals (Synthesis in THF using pyridine as base);

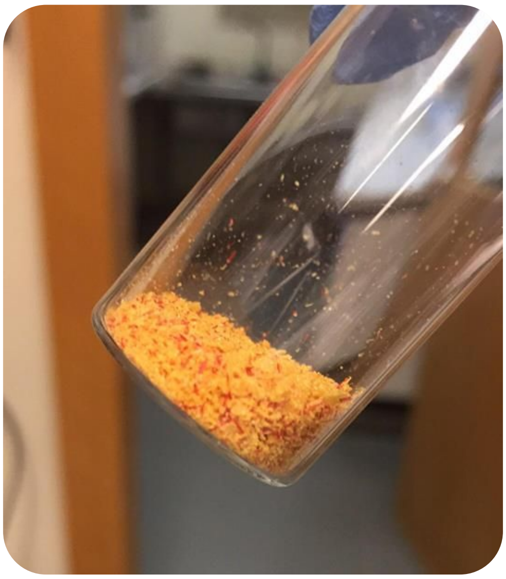

Mixture of BMTT (red crystals) and MTSB (yellow crystals), result from the synthesis in absence of base by using 1,4-dioxane as solvent.

Table S1. Summary of the single-crystal X-ray data collection and crystal structure refinement of **BMTT**.

Chemical formula	$C_{10}H_7N_3O_2S_3$
Formula weight	297.37
Color, shape	red/needle
Space group	*Pnma*
Temperature (K)	292(2)
Cell volume (Å ^{3})	1179.0(16)
Crystal system	orthorhombic
a (Å)	11.2516(19)
b (Å)	6.7170(14)
c (Å)	15.629(3)
α (deg)	90
β (deg)	90
γ (deg)	90
Z/Z´	4/1
D_c (Mg m^{-3})	1.449
Radiation (Å) (graphite monochromated)	0.71073
Max. crystal dimensions (mm)	0.45×0.35×0.07
Θ range (deg)	3.140-29.983
Range of *h, k, l*	–14,14;–8,8;–20,20
Reflections measured/independent	122800/1320
Reflections observed (I > 2 σ)	2077
Data/restraints/parameters	1536/0/112
GOF	1.075
R_1 (I >2 σ)	0.0383
wR_2	0.0844
Function minimized	$\Sigma\ w\ (\|F_o\|^2 - S\|F_c\|^2)$
Diff. density final max/min (e Å^{-3})	0.312, –0.377

Table S2. Summary of the single-crystal X-ray data collection and crystal structure refinement of **MTSB**.

Chemical formula	$C_{10}H_7N_3O_2S_3$
Formula weight	297.37
Color, shape	yellow/block
Space group	$P2_1/c$
Temperature (K)	292(2)
Cell volume (Å ^{3})	1200.3(4)
Crystal system	monoclinic
a (Å)	8.3564(14)
b (Å)	9.4051(16)
c (Å)	15.761(3)
α (deg)	90
β (deg)	104.2980(10)
ɣ (deg)	90
Z/Z'	4/1
D_c (Mg m^{-3})	1.645
Radiation (Å) (graph. monochromated)	0.71073
Max. crystal dimensions (mm)	0.35×0.33×0.27
Θ range (deg)	3.183-36.497
Range of h, k, l	–13,13;–15,15;–26,26
Reflections measured/independent	182772/5816
Reflections observed (I > 2 σ)	4050
Data/restraints/parameters	5816/0/165
GOF	1.064
R_1 (I >2 σ)	0.0349
wR_2	0.0638
Function minimized	$\Sigma\, w\, (\|F_o\|^2 - S\|F_c\|^2)$
Diff. density final max/min (e Å^{-3})	0.432, –0.540

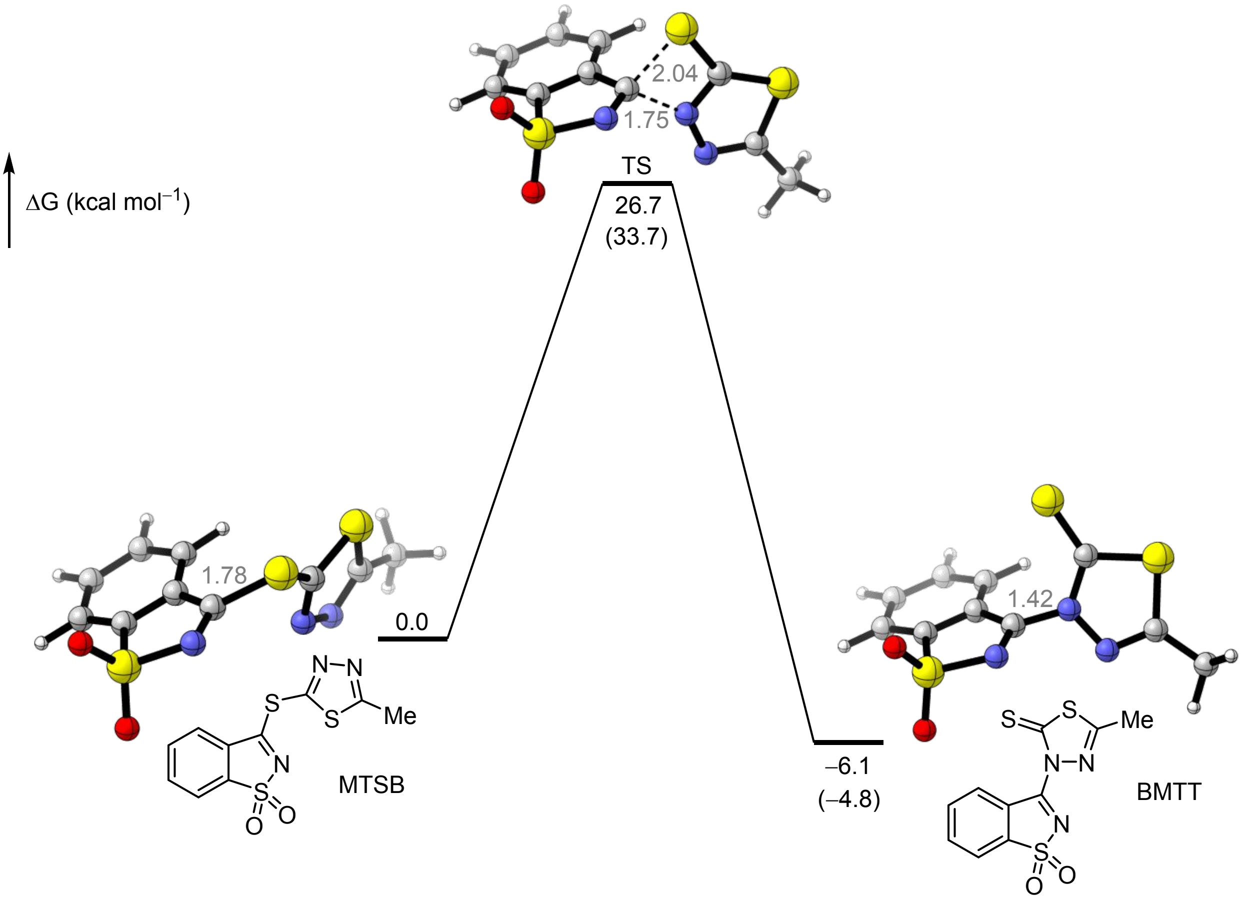

Figure S1. Gibbs free energy profile for S to N isomerization. DFT calculations were performed at the M06-2X/def2-TZVPP/PCM(THF)//M06-2X/6-31++G(d,p) or M06-2X/def2-TZVPP//M06-2X/6-31++G(d,p) (in brackets) level of theory (energy values in kcal mol^{-1}). Geometries depict the C–S and C–N bond distances in Å.

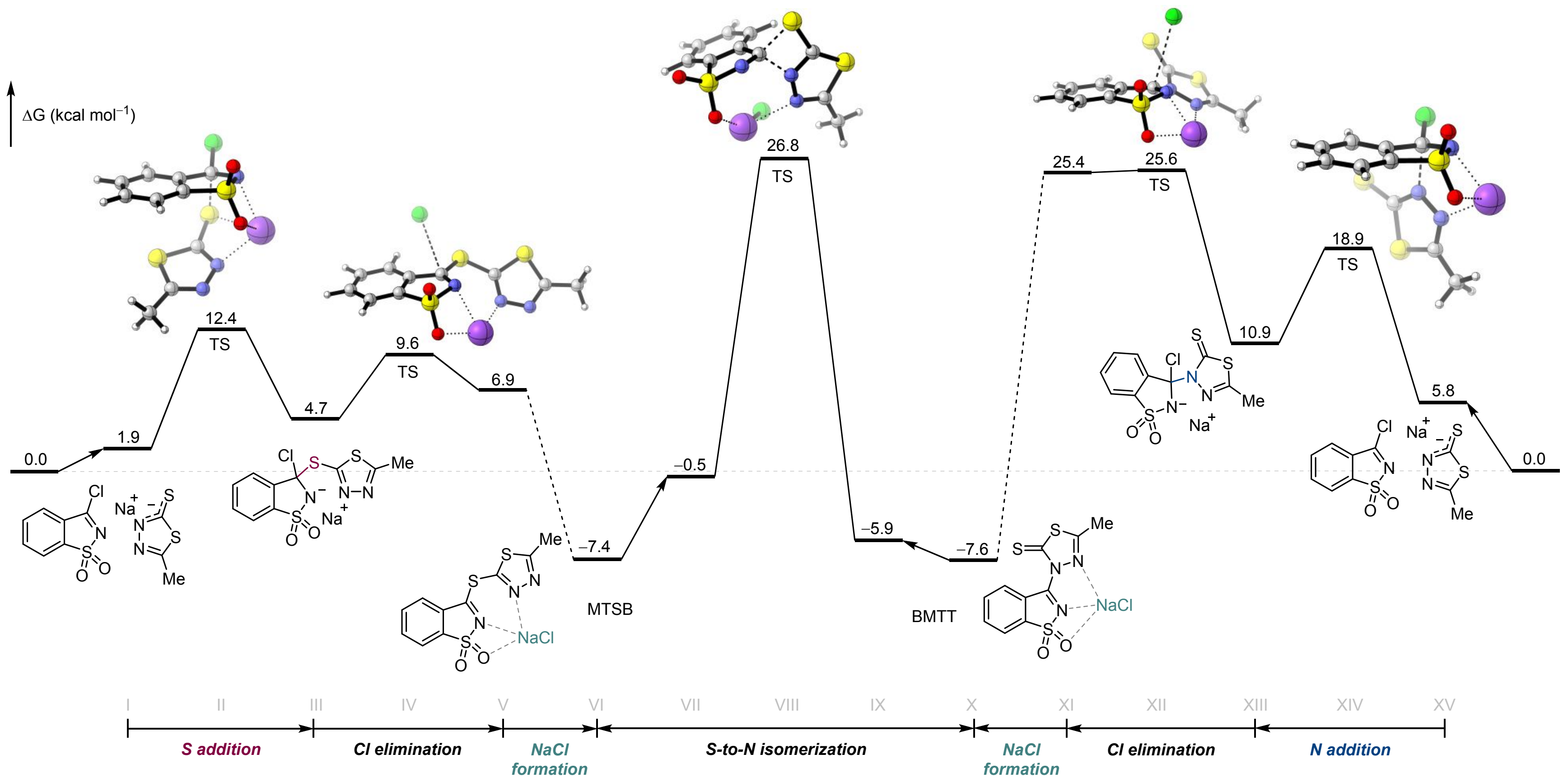

Figure S2. Complete Gibbs free energy profile for the formation of both isomers using Na^+. DFT calculations were performed at the M06-2X/def2-TZVPP/PCM(THF)//M06-2X/6-31++G(d,p) level of theory (energy values in kcal mol^{-1}).

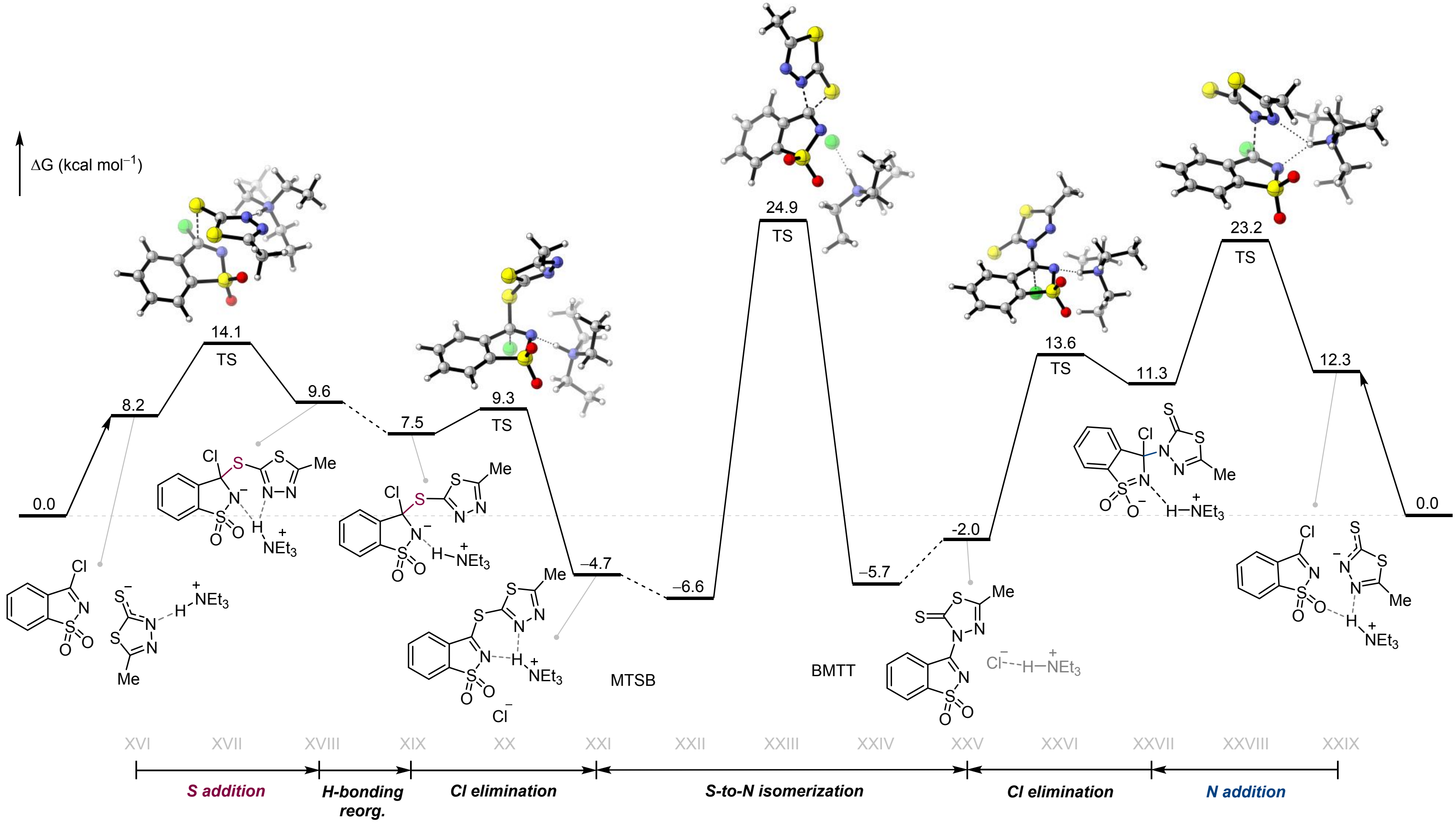

Figure S3. Complete Gibbs free energy profile for the formation of both isomers using $[Et_3NH]^+$. DFT calculations were performed at the M06-2X/def2-TZVPP/PCM(THF)//M06-2X/6-31++G(d,p) level of theory (energy values in kcal mol^{-1}).

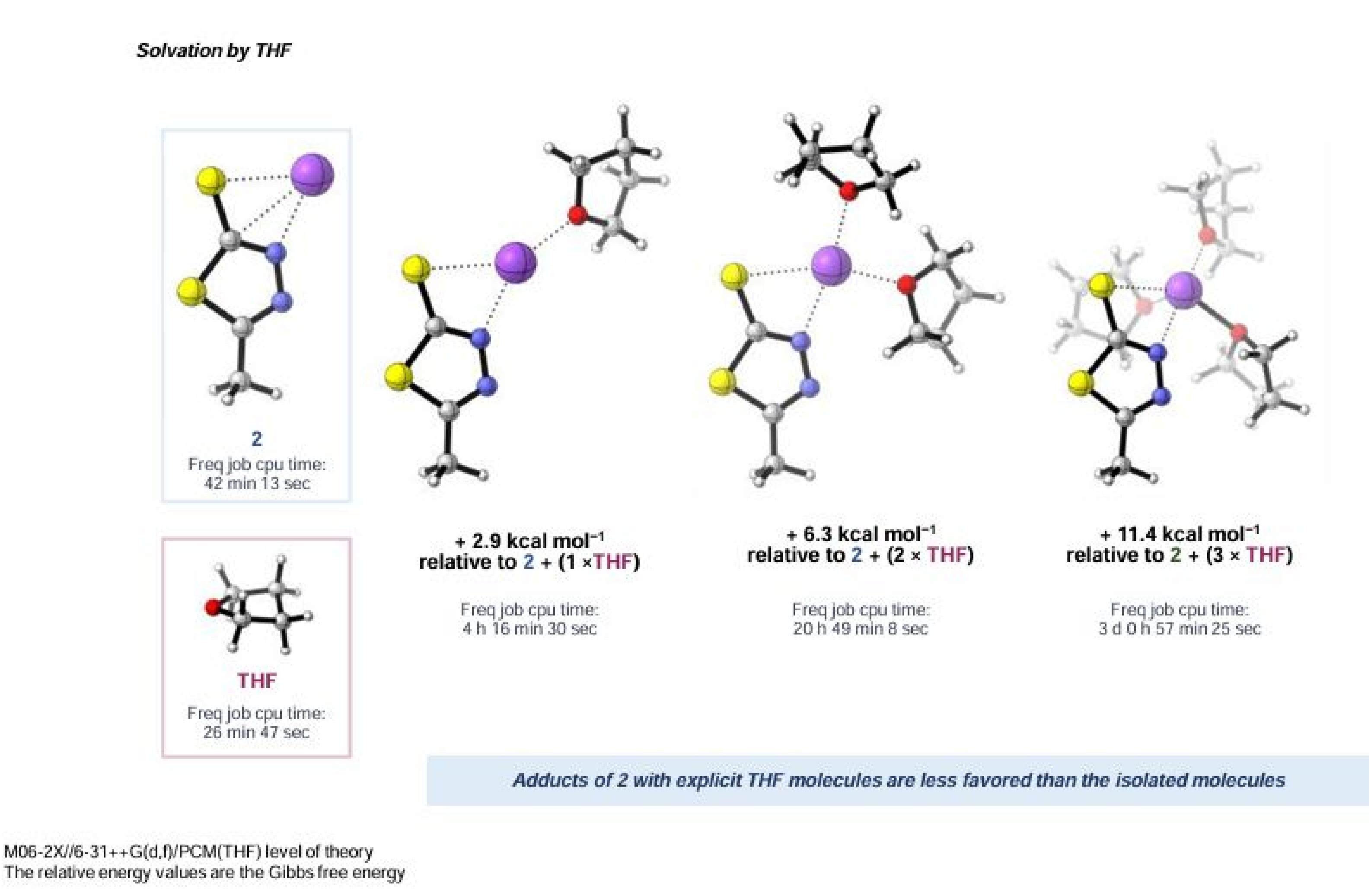

Figure S4. Gibbs free energy of adduct **2** (thiadiazole coordinated to Na^+) solvated by one, two and three THF molecules.

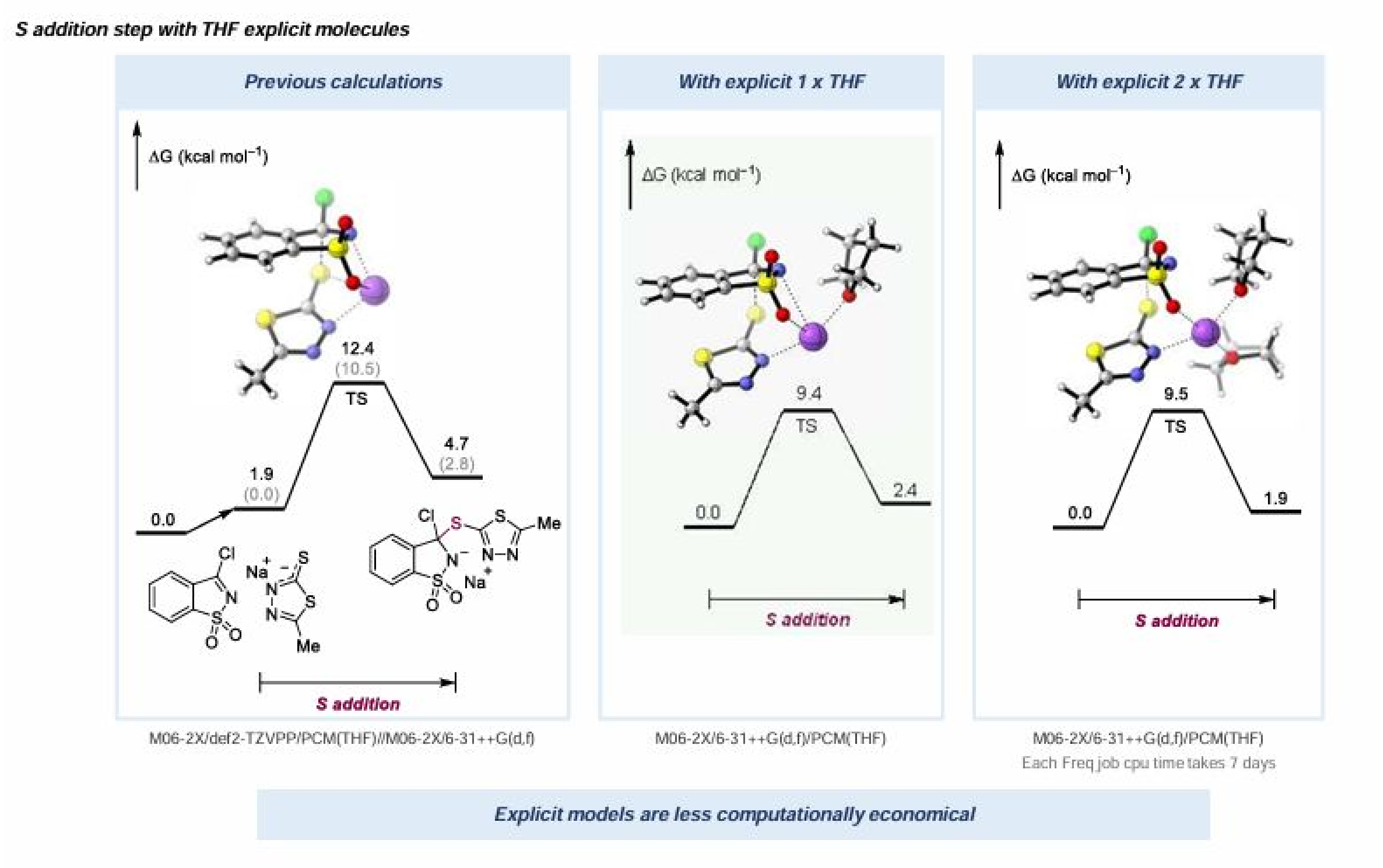

Figure S5. Gibbs free energy of S addition step with one and two THF explicit molecules.

Cartesian coordinates and Energies for Figure S2

I
SCF energy: −2516.503701 Hartree
Free energy correction: 0.117798 Hartree
Imaginary Frequency: none

16	-3.024952000	-1.231504000	0.072372000
6	-1.612175000	-0.736634000	0.997520000
7	-1.622112000	0.571405000	1.200416000
7	-2.682076000	1.228642000	0.651067000
6	-3.503518000	0.444123000	0.027488000
16	-0.398854000	-1.814143000	1.558482000
6	-0.958901000	0.178005000	-2.408154000
6	-0.905715000	1.521305000	-2.029256000
6	0.122771000	1.997663000	-1.210257000
6	1.067535000	1.075807000	-0.807233000
6	1.026602000	-0.264440000	-1.171818000
6	0.007080000	-0.737254000	-1.983398000
7	2.981461000	-0.340099000	0.194322000
6	2.164907000	-0.982877000	-0.548157000
16	2.464481000	1.279297000	0.278595000
8	3.477537000	2.154782000	-0.264659000
8	1.996065000	1.520988000	1.648729000
6	-4.729768000	0.931328000	-0.673695000
1	-1.777501000	-0.167498000	-3.031126000
1	-1.682312000	2.203700000	-2.357949000
1	0.162450000	3.033787000	-0.892632000
1	-0.050249000	-1.787779000	-2.248307000
1	-4.803694000	2.010294000	-0.527312000
1	-4.681481000	0.717928000	-1.746204000
1	-5.629909000	0.454851000	-0.276185000
17	2.405790000	-2.645263000	-0.871081000
11	0.210076000	0.597983000	2.616861000

II
SCF energy: −2516.485793 Hartree
Free energy correction: 0.118665 Hartree
Imaginary Frequency: −145.5539 cm^{-1}

16	-2.340026000	0.294489000	-1.236446000
6	-1.228125000	-1.029611000	-1.009219000
7	-1.598009000	-1.808504000	-0.018432000
7	-2.738683000	-1.421820000	0.605903000
6	-3.255122000	-0.343193000	0.096402000
16	0.217572000	-1.275963000	-1.936030000
6	-0.088203000	3.192350000	-0.813302000
6	-0.517705000	3.246880000	0.518216000
6	-0.169314000	2.243380000	1.421896000
6	0.601932000	1.198739000	0.936058000
6	1.025781000	1.126783000	-0.382782000
6	0.683380000	2.130148000	-1.281617000
7	2.077050000	-0.838962000	0.479871000
6	1.801344000	-0.134320000	-0.607416000
16	1.342675000	-0.167330000	1.813205000
8	2.300314000	0.323251000	2.786812000
8	0.342796000	-1.139482000	2.291355000
6	-4.511542000	0.281994000	0.607847000
1	-0.365592000	3.989868000	-1.494785000
1	-1.119002000	4.085424000	0.853743000
1	-0.474289000	2.280234000	2.462461000
1	1.002848000	2.071774000	-2.317454000
1	-4.870895000	-0.313341000	1.448215000
1	-4.329089000	1.305569000	0.945742000
1	-5.281181000	0.309067000	-0.167933000
17	3.033247000	-0.114948000	-1.865477000
11	0.451501000	-2.714333000	0.610747000

III
SCF energy: −2516.493408 Hartree
Free energy correction: 0.119310 Hartree
Imaginary Frequency: none

16	-2.476674000	0.933263000	-0.870849000
6	-1.487143000	-0.463607000	-1.147342000
7	-1.856941000	-1.484878000	-0.422091000
7	-2.921187000	-1.234498000	0.384079000
6	-3.371462000	-0.017293000	0.267172000
16	-0.125825000	-0.482007000	-2.262684000
6	0.635870000	3.362329000	0.201512000
6	0.667387000	3.127960000	1.582165000
6	0.912426000	1.849676000	2.080107000
6	1.090498000	0.831815000	1.153755000
6	1.055194000	1.045808000	-0.212611000
6	0.834527000	2.326483000	-0.708990000
7	1.356113000	-1.332359000	-0.139128000
6	1.255220000	-0.248829000	-0.987367000
16	1.534653000	-0.877494000	1.408341000
8	2.881437000	-0.933413000	1.961123000
8	0.508148000	-1.627241000	2.181925000
6	-4.540162000	0.497729000	1.040960000
1	0.458919000	4.368754000	-0.164127000
1	0.513042000	3.952891000	2.270103000
1	0.966441000	1.652044000	3.145636000
1	0.818980000	2.504459000	-1.780436000
1	-4.911060000	-0.308145000	1.675232000
1	-4.248297000	1.342778000	1.669276000
1	-5.339242000	0.825370000	0.371434000
17	2.707366000	-0.028917000	-2.136240000
11	-0.243200000	-2.913799000	0.473768000

IV
SCF energy: −2516.460775 Hartree
Free energy correction: 0.117863 Hartree
Imaginary Frequency: −24.8659 cm^{-1}

16	3.549310000	1.147849000	0.480595000
6	2.083069000	0.502209000	-0.175697000
7	2.264479000	-0.628249000	-0.796049000
7	3.557435000	-1.066121000	-0.774899000
6	4.359604000	-0.246217000	-0.160463000
16	0.602895000	1.503382000	-0.083440000
6	-4.050026000	1.384792000	-0.912357000
6	-4.728745000	0.186946000	-0.693904000
6	-4.054836000	-0.940613000	-0.209558000
6	-2.706259000	-0.790129000	0.036431000
6	-2.009888000	0.404591000	-0.158902000
6	-2.680361000	1.517064000	-0.656148000
7	-0.220536000	-0.953782000	0.623808000
6	-0.566950000	0.216255000	0.184526000
16	-1.555726000	-1.993772000	0.627775000
8	-1.867821000	-2.487493000	1.950164000
8	-1.213222000	-2.986135000	-0.415862000
6	5.827403000	-0.480640000	-0.019112000
1	-4.593561000	2.249266000	-1.278208000
1	-5.792660000	0.124004000	-0.897417000
1	-4.563285000	-1.882765000	-0.036392000
1	-2.167555000	2.475245000	-0.759152000
1	6.070868000	-1.431804000	-0.493679000
1	6.395877000	0.316573000	-0.504317000
1	6.118456000	-0.518481000	1.033334000
17	-0.986525000	3.970867000	0.446435000
11	0.941795000	-2.505053000	-0.918707000

V
SCF energy: −2516.463197 Hartree
Free energy correction: 0.115951 Hartree
Imaginary Frequency: none

16	3.457470000	1.010791000	0.813857000
6	2.072520000	0.495364000	-0.080415000
7	2.316602000	-0.525842000	-0.852696000
7	3.598502000	-0.979132000	-0.768313000
6	4.331086000	-0.278324000	0.048905000
16	0.568670000	1.453494000	-0.033419000
6	-4.116379000	1.494871000	-0.172738000
6	-4.792068000	0.277493000	-0.103756000
6	-4.093346000	-0.926297000	0.060471000
6	-2.720783000	-0.831868000	0.145849000
6	-2.028291000	0.380315000	0.085372000
6	-2.721044000	1.574043000	-0.080221000
7	-0.190134000	-1.087103000	0.433081000
6	-0.562828000	0.136429000	0.213382000
16	-1.528761000	-2.119452000	0.379645000
8	-1.722868000	-2.850790000	1.612158000
8	-1.283734000	-2.895601000	-0.856136000
6	5.777301000	-0.552387000	0.298767000
1	-4.677612000	2.414969000	-0.297688000
1	-5.874568000	0.256208000	-0.175768000
1	-4.603814000	-1.881345000	0.119578000
1	-2.192297000	2.538084000	-0.131180000
1	6.067368000	-1.424418000	-0.288217000
1	6.390641000	0.300691000	-0.001580000
1	5.961308000	-0.753027000	1.356869000
17	-0.789056000	4.199709000	-0.228106000
11	0.862353000	-2.260520000	-1.408353000

VI
SCF energy: −2516.509302 Hartree
Free energy correction: 0.116965 Hartree
Imaginary Frequency: none

16	-3.670404000	-1.071702000	1.029355000
6	-2.169647000	-0.712200000	0.242855000
7	-2.249765000	0.266671000	-0.607839000
7	-3.500038000	0.796632000	-0.692772000
6	-4.364151000	0.208029000	0.078760000
16	-0.733284000	-1.685574000	0.559462000
6	3.882715000	-2.156269000	0.204924000
6	4.670081000	-1.002643000	0.191083000
6	4.094885000	0.264130000	0.326035000
6	2.723874000	0.309642000	0.480155000
6	1.927358000	-0.827732000	0.495528000
6	2.497514000	-2.083898000	0.351476000
7	0.220308000	0.809781000	0.764806000
6	0.498588000	-0.434049000	0.616052000
16	1.648780000	1.722659000	0.638044000
8	1.912046000	2.459732000	1.855361000
8	1.501002000	2.453237000	-0.622238000
6	-5.806561000	0.587429000	0.143161000
1	4.354852000	-3.124696000	0.080905000
1	5.743655000	-1.089138000	0.062223000
1	4.694422000	1.167461000	0.301970000
1	1.885515000	-2.979366000	0.315571000
1	-5.966828000	1.424832000	-0.536755000
1	-6.442850000	-0.247342000	-0.161084000
1	-6.089209000	0.887242000	1.155141000
17	0.799020000	-0.696821000	-2.502607000
11	-0.364566000	1.366442000	-1.745268000

VII
SCF energy: −2516.506736 Hartree
Free energy correction: 0.118289 Hartree
Imaginary Frequency: none

16	2.933807000	0.348432000	1.142925000
6	1.737945000	-0.891803000	1.032934000
7	1.729882000	-1.508948000	-0.109937000
7	2.669987000	-1.035419000	-0.971940000
6	3.375108000	-0.055492000	-0.481137000
16	0.566768000	-1.243467000	2.321117000
6	-1.488436000	2.875605000	1.216213000
6	-2.451349000	2.882052000	0.207463000
6	-2.863572000	1.695065000	-0.407675000
6	-2.275490000	0.532610000	0.042159000
6	-1.310147000	0.506928000	1.041855000
6	-0.900901000	1.684800000	1.646932000
7	-1.368244000	-1.830126000	0.584453000
6	-0.834037000	-0.881556000	1.251274000
16	-2.461970000	-1.136666000	-0.547964000
8	-3.798267000	-1.656836000	-0.370616000
8	-1.796945000	-1.292754000	-1.845889000
6	4.367533000	0.720027000	-1.274792000
1	-1.176795000	3.814577000	1.660139000
1	-2.876158000	3.824613000	-0.120260000
1	-3.595964000	1.692950000	-1.207249000
1	-0.135701000	1.676976000	2.416187000
1	4.687962000	0.117338000	-2.125426000
1	3.866427000	1.621780000	-1.643642000
1	5.235507000	1.004768000	-0.677414000
17	0.712495000	1.603973000	-1.501418000
11	0.424095000	-0.791325000	-2.170257000

VIII
SCF energy: −2516.453464 Hartree
Free energy correction: 0.118487 Hartree
Imaginary Frequency: −203.8347 cm^{-1}

16	3.406992000	-1.062754000	0.328853000
6	1.824462000	-1.707915000	0.297216000
7	1.054049000	-0.970763000	-0.459313000
7	1.561706000	0.133801000	-1.025919000
6	2.818545000	0.243370000	-0.723972000
16	0.688444000	-2.791636000	1.035316000
6	-0.913942000	1.360996000	2.663620000
6	-2.135507000	1.828982000	2.173408000
6	-2.744492000	1.224238000	1.072825000
6	-2.094515000	0.136156000	0.513268000
6	-0.888458000	-0.343119000	0.995299000
6	-0.274145000	0.269081000	2.079321000
7	-1.303855000	-1.922960000	-0.780052000
6	-0.420437000	-1.485515000	0.105052000
16	-2.418134000	-0.743120000	-1.012420000
8	-3.765551000	-1.256014000	-1.145412000
8	-1.952641000	0.195798000	-2.066819000
6	3.687284000	1.366273000	-1.170832000
1	-0.443147000	1.870946000	3.496425000
1	-2.604268000	2.688403000	2.640168000
1	-3.682364000	1.588734000	0.666385000
1	0.681963000	-0.079323000	2.460018000
1	3.851589000	1.310114000	-2.249686000
1	3.149256000	2.292286000	-0.925737000
1	4.650994000	1.350064000	-0.660840000
17	0.879390000	3.170074000	0.007474000
11	-0.361910000	1.735781000	-1.605288000

IX
SCF energy: −2516.510104 Hartree
Free energy correction: 0.118440 Hartree
Imaginary Frequency: none

16	3.650754000	-0.764794000	-0.163882000
6	2.023626000	-1.250474000	-0.614599000
7	1.254748000	-0.111102000	-0.541223000
7	1.888152000	1.092385000	-0.328978000
6	3.133504000	0.905936000	-0.085315000
16	1.573527000	-2.742495000	-1.128049000
6	-1.981850000	-2.526562000	1.600852000
6	-3.278968000	-2.071231000	1.350571000

6	-3.503864000	-0.999087000	0.486319000
6	-2.390979000	-0.454832000	-0.126568000
6	-1.095775000	-0.920530000	0.074004000
6	-0.875394000	-1.954732000	0.976821000
7	-0.614654000	0.959438000	-1.315941000
6	-0.142055000	-0.036406000	-0.652824000
16	-2.295467000	1.026671000	-1.101053000
8	-2.997297000	0.936044000	-2.360321000
8	-2.485407000	2.210595000	-0.252437000
6	4.058881000	2.013299000	0.286994000
1	-1.827906000	-3.333067000	2.309151000
1	-4.118969000	-2.536792000	1.854932000
1	-4.497220000	-0.599144000	0.315206000
1	0.125954000	-2.292724000	1.207679000
1	4.975193000	1.978120000	-0.306297000
1	3.556703000	2.967152000	0.122077000
1	4.319611000	1.929626000	1.345777000
17	0.274302000	1.021385000	2.401972000
11	-0.325123000	2.656611000	0.639385000

X
SCF energy: −2516.508509 Hartree
Free energy correction: 0.117139 Hartree
Imaginary Frequency: none

6	-3.638790000	1.513759000	-1.502902000
6	-4.472773000	0.414349000	-1.284153000
6	-4.006527000	-0.715236000	-0.607199000
6	-2.702517000	-0.673712000	-0.152795000
6	-1.867550000	0.425751000	-0.326501000
6	-2.326306000	1.534365000	-1.031363000
7	-0.316498000	-1.063850000	0.690442000
6	-0.516194000	0.106689000	0.205098000
7	0.596827000	0.953384000	0.058702000
6	0.668372000	2.271739000	0.468651000
16	2.311647000	2.788540000	0.131896000
6	2.746479000	1.161450000	-0.359910000
7	1.768672000	0.323802000	-0.319447000
16	-0.488548000	3.167091000	1.208719000
16	-1.764140000	-1.978146000	0.618106000
8	-2.219132000	-2.252693000	1.961262000
8	-1.498780000	-3.056499000	-0.317028000
6	4.124938000	0.808723000	-0.796803000
1	-4.017995000	2.370648000	-2.048778000
1	-5.490864000	0.430354000	-1.658255000
1	-4.630619000	-1.589449000	-0.457191000
1	-1.690970000	2.395643000	-1.198530000
1	4.224584000	-0.281983000	-0.852518000
1	4.321830000	1.253836000	-1.776736000
1	4.855536000	1.201954000	-0.085861000
11	1.747358000	-2.180718000	0.017416000
17	4.094689000	-2.678821000	-0.428691000

XI
SCF energy: −2516.442311 Hartree
Free energy correction: 0.118494 Hartree
Imaginary Frequency: none

6	2.320418000	-2.883076000	-1.351767000
6	3.613990000	-2.414891000	-1.110817000
6	3.815409000	-1.193287000	-0.466144000
6	2.687061000	-0.494662000	-0.080878000
6	1.383857000	-0.948430000	-0.296112000
6	1.193236000	-2.164466000	-0.949668000
7	0.955321000	1.083706000	0.894824000
6	0.431083000	0.100031000	0.172792000
7	-0.825019000	0.211162000	-0.267861000
6	-1.728554000	-0.869864000	-0.542771000
16	-3.003122000	-0.120803000	-1.547393000
6	-2.509698000	1.445800000	-0.961969000
7	-1.386070000	1.497925000	-0.327290000
16	-2.020204000	-2.062275000	0.544340000
16	2.595041000	1.088395000	0.728687000
8	3.324481000	1.102738000	1.980383000
8	2.847412000	2.206311000	-0.214639000
6	-3.331893000	2.666273000	-1.210681000
1	2.183749000	-3.836175000	-1.851669000
1	4.468691000	-3.005301000	-1.423418000
1	4.809508000	-0.805094000	-0.271380000
1	0.196278000	-2.547429000	-1.124593000
1	-2.785318000	3.563866000	-0.915434000
1	-3.602215000	2.735069000	-2.267572000
1	-4.254794000	2.608077000	-0.627688000
17	-3.287889000	-1.202031000	2.271310000
11	0.536050000	3.068538000	-0.106932000

XII
SCF energy: −2516.435077 Hartree
Free energy correction: 0.119597 Hartree
Imaginary Frequency: −72.5082 cm^{-1}

6	2.446450000	-2.529912000	-1.663604000
6	3.679919000	-1.950595000	-1.359888000
6	3.740459000	-0.752582000	-0.645914000
6	2.538417000	-0.203434000	-0.244161000
6	1.293558000	-0.784037000	-0.495602000
6	1.244261000	-1.958543000	-1.243695000
7	0.622211000	1.165524000	0.706360000
6	0.223362000	0.119908000	0.015068000
7	-1.067119000	0.087568000	-0.422362000
6	-1.926499000	-1.064534000	-0.431358000
16	-3.548850000	-0.448617000	-0.835405000
6	-2.974314000	1.182624000	-0.678302000
7	-1.712352000	1.322888000	-0.466415000
16	-1.601831000	-2.440345000	0.342774000
16	2.264286000	1.346422000	0.580721000
8	2.959352000	1.441314000	1.845141000
8	2.397320000	2.489842000	-0.357867000
6	-3.889623000	2.354147000	-0.815439000
1	2.417287000	-3.453305000	-2.232213000
1	4.596762000	-2.428549000	-1.688006000
1	4.683615000	-0.267847000	-0.417532000
1	0.300315000	-2.430653000	-1.479473000
1	-3.321984000	3.284786000	-0.758468000
1	-4.420419000	2.315477000	-1.770113000
1	-4.630973000	2.341953000	-0.012481000
17	-1.176387000	-1.451891000	2.667547000
11	0.049800000	3.094690000	-0.331214000

XIII
SCF energy: −2516.476988 Hartree
Free energy correction: 0.121111 Hartree
Imaginary Frequency: none

6	1.829473000	-2.784713000	-1.619361000
6	2.952303000	-2.042200000	-1.996275000
6	3.165699000	-0.774100000	-1.461184000
6	2.207948000	-0.287826000	-0.585408000
6	1.077192000	-0.997499000	-0.212152000
6	0.890830000	-2.282172000	-0.718009000
7	0.795017000	1.044546000	1.028906000
6	0.219518000	-0.192111000	0.777735000
7	-1.195384000	0.021177000	0.219623000
6	-2.206881000	-0.901001000	0.098108000
16	-3.587738000	-0.061840000	-0.623781000
6	-2.656593000	1.392747000	-0.719094000
7	-1.460102000	1.280372000	-0.254074000
16	-2.320966000	-2.498698000	0.476923000
16	2.251603000	1.211452000	0.361410000
8	3.390610000	1.187809000	1.268301000

8	2.187528000	2.427287000	-0.506796000
6	-3.194444000	2.664652000	-1.289895000
1	1.689758000	-3.782465000	-2.022759000
1	3.670742000	-2.463665000	-2.691706000
1	4.046238000	-0.188469000	-1.703997000
1	0.045819000	-2.881689000	-0.404468000
1	-2.412787000	3.427636000	-1.313546000
1	-3.549691000	2.508856000	-2.311478000
1	-4.030505000	3.034505000	-0.690516000
17	0.017743000	-1.141240000	2.341092000
11	0.130938000	3.019423000	0.109732000

XIV
SCF energy: −2516.467056 Hartree
Free energy correction: 0.118531 Hartree
Imaginary Frequency: −160.2141 cm^{-1}

6	-1.533116000	2.951130000	-1.412436000
6	-2.322967000	2.159507000	-2.249364000
6	-2.578383000	0.821937000	-1.937037000
6	-1.983572000	0.327902000	-0.790742000
6	-1.175477000	1.095290000	0.039686000
6	-0.954962000	2.434859000	-0.250079000
7	-1.223383000	-0.922412000	1.311541000
6	-0.682070000	0.271027000	1.190025000
7	1.226678000	-0.033914000	0.409855000
6	2.174773000	0.843129000	0.058986000
16	3.401249000	-0.017305000	-0.915931000
6	2.490278000	-1.473530000	-0.723325000
7	1.412283000	-1.307565000	-0.018585000
16	2.319487000	2.469357000	0.416178000
16	-2.223910000	-1.238595000	0.006593000
8	-3.607345000	-1.394081000	0.416373000
8	-1.622663000	-2.380359000	-0.716300000
6	2.901863000	-2.790479000	-1.298429000
1	-1.365294000	3.992306000	-1.666617000
1	-2.760547000	2.592328000	-3.142802000
1	-3.222604000	0.203506000	-2.552590000
1	-0.335249000	3.042224000	0.398157000
1	2.110717000	-3.528564000	-1.140154000
1	3.073035000	-2.706892000	-2.374671000
1	3.824313000	-3.152981000	-0.836298000
17	-0.177352000	1.071885000	2.643566000
11	-0.031433000	-2.898287000	0.816450000

XV
SCF energy: −2516.488911 Hartree
Free energy correction: 0.115816 Hartree
Imaginary Frequency: none

6	-0.556383000	0.935290000	2.458277000
6	-0.768695000	-0.442889000	2.528154000
6	0.031446000	-1.340070000	1.811675000
6	1.021236000	-0.782450000	1.027835000
6	1.237865000	0.586953000	0.936834000
6	0.455201000	1.475172000	1.659172000
7	2.896614000	-0.128196000	-0.622366000
6	2.324118000	0.854004000	-0.034885000
7	-1.277766000	-0.091819000	-1.188403000
6	-1.730546000	1.092931000	-0.792923000
16	-3.254127000	0.865726000	0.107833000
6	-3.119397000	-0.844514000	-0.155776000
7	-2.060394000	-1.162810000	-0.834186000
16	-1.019133000	2.595867000	-1.054651000
16	2.205283000	-1.569769000	-0.043043000
8	3.189990000	-2.335653000	0.687894000
8	1.533134000	-2.223857000	-1.174556000
6	-4.108882000	-1.847974000	0.341265000
1	-1.206143000	1.603196000	3.013219000
1	-1.574196000	-0.826949000	3.146178000
1	-0.128046000	-2.411648000	1.858505000
1	0.599319000	2.545273000	1.571066000
1	-3.787795000	-2.843517000	0.028858000
1	-4.178118000	-1.823541000	1.432998000
1	-5.106143000	-1.651775000	-0.062016000
17	2.816580000	2.457959000	-0.347723000
11	-0.256642000	-1.691672000	-2.319046000

Cartesian coordinates and Energies for Figure S3

XVI

SCF energy: −2646.847682 Hartree
Free energy correction: 0.327503 Hartree
Imaginary Frequency: none

16	-0.897352000	2.282527000	-1.415686000
6	0.236868000	0.931792000	-1.455482000
7	1.285399000	1.214007000	-0.693761000
7	1.277345000	2.441724000	-0.103065000
6	0.210125000	3.122050000	-0.363137000
16	-0.006398000	-0.478007000	-2.370991000
6	-4.399714000	-0.559101000	-1.345790000
6	-4.621322000	0.518086000	-0.480823000
6	-3.752642000	0.778926000	0.580501000
6	-2.675573000	-0.075885000	0.727771000
6	-2.448212000	-1.151799000	-0.117352000
6	-3.309744000	-1.410771000	-1.174883000
7	-0.562084000	-1.434685000	1.314881000
6	-1.223997000	-1.869697000	0.316176000
16	-1.376939000	-0.061385000	1.951892000
8	-0.522602000	1.102857000	1.810606000
8	-1.895199000	-0.422726000	3.257401000
6	-0.062545000	4.471366000	0.214621000
1	-5.083993000	-0.728828000	-2.170056000
1	-5.477206000	1.165259000	-0.641140000
1	-3.908348000	1.615015000	1.253747000
1	-3.116284000	-2.230945000	-1.857538000
1	0.827700000	4.803382000	0.751345000
1	-0.309195000	5.199428000	-0.562574000
1	-0.898507000	4.420446000	0.918887000
17	-0.786208000	-3.352349000	-0.442275000
1	2.289259000	0.155225000	-0.182015000
7	3.090834000	-0.584266000	0.055740000
6	4.143102000	-0.245104000	-0.944856000
1	4.968601000	-0.954770000	-0.832341000
1	3.676464000	-0.397951000	-1.923636000
6	4.633546000	1.189271000	-0.809274000
1	5.181345000	1.345597000	0.124057000
1	3.799437000	1.894695000	-0.852388000
1	5.315441000	1.407035000	-1.634701000
6	2.485969000	-1.929498000	-0.198209000
1	1.690960000	-2.036372000	0.544261000
1	2.003586000	-1.871583000	-1.178224000
6	3.476941000	-3.080543000	-0.115162000
1	4.032564000	-3.083046000	0.827992000
1	4.192096000	-3.071514000	-0.941156000
1	2.921388000	-4.019740000	-0.173262000
6	3.527822000	-0.416259000	1.480808000
1	3.161394000	-1.283632000	2.034909000
1	4.622091000	-0.446702000	1.508728000
6	2.975239000	0.858449000	2.102245000
1	1.881127000	0.857626000	2.086545000
1	3.303337000	1.752957000	1.569212000
1	3.310054000	0.921988000	3.141052000

XVII

SCF energy: −2646.839505 Hartree
Free energy correction: 0.329324 Hartree
Imaginary Frequency: −115.1102 cm^{-1}

16	-0.964117000	2.297767000	-1.559947000
6	0.088564000	0.903671000	-1.552626000
7	1.213857000	1.168849000	-0.922409000
7	1.316155000	2.436013000	-0.442501000
6	0.260381000	3.153126000	-0.678216000
16	-0.322709000	-0.621498000	-2.221563000
6	-4.700846000	-0.775976000	-0.714952000
6	-4.871097000	0.176406000	0.298760000
6	-3.810977000	0.524309000	1.133349000
6	-2.599146000	-0.111002000	0.909372000
6	-2.413727000	-1.047063000	-0.092797000
6	-3.472673000	-1.397908000	-0.923305000
7	-0.221993000	-1.070458000	0.867702000
6	-0.998568000	-1.535855000	-0.077862000
16	-1.054924000	0.068542000	1.782838000
8	-0.529577000	1.416897000	1.585592000
8	-1.154858000	-0.416446000	3.151847000
6	0.098128000	4.551885000	-0.184421000
1	-5.541598000	-1.030027000	-1.352129000
1	-5.839724000	0.645514000	0.436411000
1	-3.924499000	1.253449000	1.928721000
1	-3.331211000	-2.122014000	-1.719070000
1	1.061273000	4.902106000	0.189584000
1	-0.250490000	5.220692000	-0.974859000
1	-0.626125000	4.571377000	0.635275000
17	-0.765544000	-3.222964000	-0.574907000
1	2.225395000	0.017649000	-0.245437000
7	3.005857000	-0.659739000	0.054433000
6	4.242864000	-0.111856000	-0.579096000
1	5.059217000	-0.808934000	-0.371820000
1	4.042342000	-0.123839000	-1.654609000
6	4.602064000	1.296027000	-0.129485000
1	5.023874000	1.304301000	0.877452000
1	3.733026000	1.960089000	-0.162547000
1	5.362986000	1.688193000	-0.808957000
6	2.629688000	-1.977634000	-0.557118000
1	1.672541000	-2.240011000	-0.108459000
1	2.448157000	-1.787717000	-1.618432000
6	3.653222000	-3.081224000	-0.342057000
1	3.877657000	-3.229591000	0.718054000
1	4.587891000	-2.904878000	-0.879550000
1	3.228668000	-4.014306000	-0.719897000
6	3.034467000	-0.732703000	1.554925000
1	2.324727000	-1.518626000	1.821770000
1	4.039625000	-1.051147000	1.851713000
6	2.610925000	0.561617000	2.239081000
1	1.717417000	0.983804000	1.771982000
1	3.390016000	1.323329000	2.237453000
1	2.359824000	0.332806000	3.278109000

XVIII

SCF energy: −2646.845293 Hartree
Free energy correction: 0.330144 Hartree
Imaginary Frequency: none

16	-1.406896000	2.216866000	-1.321878000
6	-0.101179000	1.108280000	-1.584608000
7	1.041204000	1.554614000	-1.142766000
7	0.959659000	2.786499000	-0.580589000
6	-0.251774000	3.265952000	-0.574232000
16	-0.290440000	-0.518240000	-2.212723000
6	-4.597298000	-1.027797000	-0.429420000
6	-4.724825000	-0.447815000	0.839398000
6	-3.596973000	-0.164569000	1.606742000
6	-2.362297000	-0.489196000	1.063583000
6	-2.220314000	-1.062846000	-0.185481000
6	-3.345687000	-1.340667000	-0.954944000
7	0.074782000	-0.889085000	0.482958000
6	-0.757111000	-1.292510000	-0.521340000
16	-0.727850000	-0.180126000	1.711304000
8	-0.517369000	1.277207000	1.721698000
8	-0.483043000	-0.858086000	2.984058000
6	-0.614827000	4.557438000	0.077833000
1	-5.488918000	-1.238537000	-1.011152000
1	-5.711869000	-0.215965000	1.226314000
1	-3.672713000	0.290581000	2.588908000
1	-3.242934000	-1.792980000	-1.936772000

1	0.301232000	5.114035000	0.279472000
1	-1.275786000	5.156744000	-0.552017000
1	-1.120482000	4.352224000	1.025854000
17	-0.581841000	-3.124791000	-0.973915000
1	1.941334000	-0.177508000	-0.072505000
7	2.888315000	-0.586484000	0.068984000
6	3.866982000	0.177565000	-0.770046000
1	4.775870000	-0.426604000	-0.823315000
1	3.408799000	0.217421000	-1.761262000
6	4.195047000	1.580683000	-0.290580000
1	4.797007000	1.568538000	0.620461000
1	3.295836000	2.179151000	-0.132056000
1	4.789124000	2.063080000	-1.071443000
6	2.755211000	-2.001216000	-0.431799000
1	1.863601000	-2.395583000	0.056259000
1	2.538927000	-1.925706000	-1.501312000
6	3.964443000	-2.882086000	-0.162382000
1	4.158136000	-2.995008000	0.906937000
1	4.875797000	-2.529164000	-0.650860000
1	3.745284000	-3.875913000	-0.559664000
6	3.100450000	-0.557520000	1.557953000
1	2.490928000	-1.381714000	1.937339000
1	4.154712000	-0.783476000	1.743118000
6	2.651618000	0.744930000	2.219815000
1	1.824390000	1.212525000	1.676519000
1	3.459018000	1.470712000	2.312891000
1	2.273763000	0.515052000	3.218505000

XIX

SCF energy: −2646.850068 Hartree

Free energy correction: 0.329696 Hartree

Imaginary Frequency: none

16	2.660008000	1.604321000	0.530387000
6	1.172241000	1.439567000	1.413010000
7	0.335630000	2.412277000	1.199000000
7	0.808065000	3.339268000	0.326603000
6	1.994490000	3.057775000	-0.132764000
16	0.769783000	0.032422000	2.383134000
6	3.572654000	-2.881892000	0.063290000
6	3.593544000	-2.630861000	-1.314735000
6	2.569825000	-1.904616000	-1.918518000
6	1.542374000	-1.457547000	-1.100065000
6	1.507070000	-1.694887000	0.261590000
6	2.533129000	-2.415614000	0.864521000
7	-0.482553000	-0.406269000	-0.016639000
6	0.289769000	-1.062275000	0.914747000
16	0.127846000	-0.463832000	-1.526757000
8	0.560643000	0.859406000	-1.989397000
8	-0.784989000	-1.171910000	-2.436678000
6	2.681946000	3.876884000	-1.173388000
1	4.381831000	-3.447353000	0.514017000
1	4.416923000	-3.002774000	-1.915724000
1	2.569790000	-1.691755000	-2.982531000
1	2.518084000	-2.602744000	1.933977000
1	2.665271000	3.342986000	-2.127894000
1	2.140988000	4.817623000	-1.283545000
1	3.720351000	4.082440000	-0.903837000
17	-0.656663000	-2.432734000	1.796337000
1	-2.054511000	-0.049734000	0.046526000
7	-3.108208000	0.078600000	-0.179045000
6	-3.812185000	0.595398000	1.041712000
1	-4.405754000	1.465171000	0.747112000
1	-4.506394000	-0.179469000	1.376342000
6	-2.815338000	0.935013000	2.139255000
1	-2.059698000	1.650238000	1.798466000
1	-2.291018000	0.034222000	2.472834000
1	-3.343626000	1.360603000	2.996238000
6	-3.534808000	-1.300588000	-0.576384000
1	-2.791385000	-1.646131000	-1.301260000
1	-3.432566000	-1.913040000	0.324387000
6	-4.944754000	-1.363306000	-1.141468000
1	-5.014562000	-0.869023000	-2.113018000
1	-5.680640000	-0.914058000	-0.467040000
1	-5.221265000	-2.410226000	-1.284700000
6	-3.127061000	1.038880000	-1.328751000
1	-2.641094000	0.521080000	-2.162281000
1	-4.173999000	1.223866000	-1.588778000
6	-2.392470000	2.328992000	-0.997636000
1	-2.379010000	2.962096000	-1.887699000
1	-1.355873000	2.135311000	-0.711708000
1	-2.879106000	2.887937000	-0.193159000

XX

SCF energy: −2646.844125 Hartree

Free energy correction: 0.331144 Hartree

Imaginary Frequency: −63.5782 cm^{-1}

16	2.989017000	-1.475552000	-0.242971000
6	1.518958000	-1.655837000	-1.147209000
7	0.915763000	-2.787916000	-0.943423000
7	1.573665000	-3.586202000	-0.059504000
6	2.660955000	-3.046375000	0.413158000
16	0.837961000	-0.386709000	-2.168254000
6	2.564851000	3.826064000	-0.846415000
6	2.504945000	4.126689000	0.519681000
6	1.768956000	3.327260000	1.393598000
6	1.109573000	2.239476000	0.844563000
6	1.153619000	1.932029000	-0.505497000
6	1.890675000	2.727013000	-1.374903000
7	-0.143343000	0.093537000	0.275924000
6	0.344976000	0.698183000	-0.790760000
16	0.119035000	0.997663000	1.651000000
8	0.908619000	0.225278000	2.602089000
8	-1.147818000	1.554664000	2.135495000
6	3.528721000	-3.710613000	1.430696000
1	3.144187000	4.463685000	-1.506109000
1	3.039706000	4.989370000	0.903119000
1	1.717804000	3.539507000	2.456293000
1	1.920739000	2.499376000	-2.435639000
1	3.536619000	-3.136113000	2.360639000
1	3.124313000	-4.704479000	1.626451000
1	4.557061000	-3.803818000	1.073008000
17	-1.316929000	1.698600000	-1.975782000
1	-2.003753000	-0.237204000	-0.129544000
7	-2.976328000	-0.504266000	0.128712000
6	-3.554610000	-1.323977000	-0.995713000
1	-4.146462000	-2.123584000	-0.540826000
1	-4.234630000	-0.670704000	-1.546797000
6	-2.477734000	-1.855932000	-1.928188000
1	-1.699243000	-2.415908000	-1.403239000
1	-2.001752000	-1.027214000	-2.457683000
1	-2.941026000	-2.517952000	-2.664552000
6	-3.708485000	0.796502000	0.335844000
1	-3.097496000	1.374931000	1.032383000
1	-3.679147000	1.301732000	-0.631044000
6	-5.130016000	0.609679000	0.840408000
1	-5.162724000	0.248394000	1.870447000
1	-5.709063000	-0.071671000	0.209342000
1	-5.629720000	1.580670000	0.819411000
6	-2.834887000	-1.271489000	1.413760000
1	-2.413910000	-0.561487000	2.131619000
1	-3.845416000	-1.539026000	1.733058000
6	-1.956359000	-2.505350000	1.283947000
1	-1.830993000	-2.938659000	2.279078000
1	-0.967727000	-2.249926000	0.895666000
1	-2.405311000	-3.268062000	0.642364000

XXI
SCF energy: −2646.859096 Hartree
Free energy correction: 0.326906 Hartree
Imaginary Frequency: none

16	2.152878000	2.694049000	0.778436000
6	1.596287000	2.070061000	-0.740677000
7	2.516970000	1.476452000	-1.439373000
7	3.719881000	1.457433000	-0.808952000
6	3.701812000	2.045136000	0.354899000
16	-0.029628000	2.279491000	-1.382420000
6	-4.535751000	1.279454000	-1.585642000
6	-5.121165000	0.588128000	-0.522146000
6	-4.363697000	0.199548000	0.586128000
6	-3.024548000	0.536633000	0.573756000
6	-2.429029000	1.222443000	-0.472751000
6	-3.178896000	1.604008000	-1.576296000
7	-0.501926000	0.928085000	0.899325000
6	-0.974477000	1.390973000	-0.199293000
16	-1.759397000	0.191790000	1.781792000
8	-1.978828000	0.948775000	3.001441000
8	-1.496788000	-1.237845000	1.882603000
6	4.894795000	2.115174000	1.251113000
1	-5.143837000	1.556979000	-2.439901000
1	-6.177189000	0.342343000	-0.560070000
1	-4.803319000	-0.345903000	1.414180000
1	-2.721714000	2.109370000	-2.421042000
1	4.717084000	1.562027000	2.177548000
1	5.740467000	1.670128000	0.725296000
1	5.135283000	3.149019000	1.510107000
17	-0.874499000	-1.245805000	-1.813859000
1	0.392359000	-2.092711000	-0.719125000
7	0.951726000	-2.848465000	-0.173110000
6	2.055585000	-3.327088000	-1.071779000
1	2.902988000	-3.603410000	-0.435163000
1	1.697228000	-4.236890000	-1.560198000
6	2.447479000	-2.302285000	-2.130578000
1	2.777412000	-1.354376000	-1.700796000
1	1.601106000	-2.088010000	-2.786038000
1	3.268209000	-2.713459000	-2.724683000
6	-0.106042000	-3.883528000	0.062470000
1	-0.916077000	-3.362847000	0.580484000
1	-0.476497000	-4.152551000	-0.929756000
6	0.380922000	-5.096917000	0.838111000
1	0.627694000	-4.851383000	1.873545000
1	1.254420000	-5.561565000	0.370258000
1	-0.417474000	-5.842201000	0.859855000
6	1.438492000	-2.269470000	1.114038000
1	0.542864000	-2.011950000	1.683054000
1	1.985711000	-3.056983000	1.641943000
6	2.305218000	-1.039301000	0.914307000
1	2.478317000	-0.573871000	1.888939000
1	1.781118000	-0.316547000	0.284308000
1	3.275159000	-1.271598000	0.466396000

XXII
SCF energy: −2646.862068 Hartree
Free energy correction: 0.327013 Hartree
Imaginary Frequency: none

16	-2.894003000	-0.243222000	0.532183000
6	-2.737964000	1.478893000	0.376486000
7	-3.736280000	2.058136000	-0.214922000
7	-4.692511000	1.175812000	-0.608218000
6	-4.401982000	-0.057338000	-0.301609000
16	-1.418095000	2.406316000	1.079414000
6	3.097575000	3.112028000	1.773945000
6	4.001227000	2.696252000	0.792626000
6	3.556650000	2.056176000	-0.367778000
6	2.193368000	1.868272000	-0.492020000
6	1.283632000	2.282360000	0.467371000
6	1.725071000	2.906263000	1.626321000
7	-0.249960000	1.296207000	-1.070924000
6	-0.098172000	1.913760000	0.044615000
16	1.268553000	1.070851000	-1.793154000
8	1.338227000	1.823233000	-3.030760000
8	1.570552000	-0.358108000	-1.827316000
6	-5.290154000	-1.207348000	-0.651199000
1	3.470268000	3.595217000	2.670766000
1	5.062946000	2.868944000	0.934733000
1	4.247340000	1.724874000	-1.136141000
1	1.029515000	3.204603000	2.404180000
1	-6.176984000	-0.812851000	-1.148838000
1	-4.781213000	-1.901542000	-1.325565000
1	-5.594202000	-1.756943000	0.243246000
17	0.277979000	-0.647722000	1.833239000
1	0.769435000	-2.001329000	0.636560000
7	1.213085000	-2.891637000	0.206313000
6	0.922752000	-3.964556000	1.203856000
1	1.413270000	-4.880998000	0.863039000
1	1.392759000	-3.631783000	2.133504000
6	-0.563132000	-4.190469000	1.430606000
1	-1.049097000	-4.630716000	0.556317000
1	-1.054424000	-3.249844000	1.695151000
1	-0.683156000	-4.887623000	2.263396000
6	2.674049000	-2.561887000	0.170503000
1	2.758915000	-1.694849000	-0.486526000
1	2.930612000	-2.239792000	1.183630000
6	3.552887000	-3.710530000	-0.300028000
1	3.238266000	-4.087709000	-1.277582000
1	3.570611000	-4.542674000	0.407636000
1	4.577713000	-3.346762000	-0.404058000
6	0.634511000	-3.143368000	-1.155417000
1	1.343651000	-2.741672000	-1.880776000
1	0.569043000	-4.227938000	-1.295393000
6	-0.708802000	-2.454080000	-1.348614000
1	-1.032415000	-2.599296000	-2.382788000
1	-0.610873000	-1.380602000	-1.169103000
1	-1.480575000	-2.847769000	-0.684652000

XXIII
SCF energy: −2646.807188 Hartree
Free energy correction: 0.32 Hartree 7946
Imaginary Frequency: −280.7034 cm^{-1}

16	-4.493100000	-2.046735000	0.336461000
6	-2.886346000	-1.454467000	0.297758000
7	-2.834641000	-0.312783000	-0.340863000
7	-3.973525000	0.197652000	-0.842310000
6	-4.960355000	-0.597425000	-0.580111000
16	-1.263154000	-1.725321000	0.831965000
6	-1.161486000	2.691409000	2.546149000
6	-0.335210000	3.606364000	1.885439000
6	0.176340000	3.321911000	0.618685000
6	-0.166692000	2.103152000	0.059180000
6	-0.985093000	1.193007000	0.700737000
6	-1.496196000	1.471791000	1.959711000
7	-0.464670000	-0.046304000	-1.285836000
6	-1.153204000	-0.030154000	-0.163572000
16	0.282186000	1.400062000	-1.524710000
8	-0.326832000	2.174902000	-2.599272000
8	1.734678000	1.202551000	-1.608547000
6	-6.370095000	-0.351890000	-1.002889000
1	-1.536973000	2.930128000	3.535773000
1	-0.086067000	4.546785000	2.366296000
1	0.817083000	4.021749000	0.092146000
1	-2.110370000	0.746253000	2.485342000
1	-6.404277000	0.601753000	-1.530779000
1	-6.719416000	-1.142939000	-1.671280000
1	-7.036537000	-0.308213000	-0.137845000
17	1.464338000	-0.827449000	1.738108000

1	2.845780000	-0.765622000	0.434892000
7	3.815974000	-0.709233000	-0.026845000
6	4.669148000	-1.634467000	0.777087000
1	5.692284000	-1.556260000	0.398159000
1	4.636484000	-1.245967000	1.798841000
6	4.189780000	-3.076526000	0.759396000
1	4.311971000	-3.537395000	-0.223765000
1	3.142408000	-3.133589000	1.067236000
1	4.790847000	-3.647228000	1.471766000
6	4.220503000	0.719071000	0.183356000
1	3.464547000	1.311988000	-0.332740000
1	4.117056000	0.901640000	1.256761000
6	5.621717000	1.039731000	-0.313702000
1	5.747000000	0.774391000	-1.367565000
1	6.401351000	0.542730000	0.268651000
1	5.783139000	2.116738000	-0.227089000
6	3.698450000	-1.082592000	-1.477177000
1	3.629005000	-0.148946000	-2.037278000
1	4.622207000	-1.599158000	-1.761138000
6	2.455278000	-1.914431000	-1.759434000
1	2.396739000	-2.096217000	-2.836042000
1	1.556753000	-1.368247000	-1.460709000
1	2.466376000	-2.877684000	-1.247093000

XXIV
SCF energy: −2646.864565 Hartree
Free energy correction: 0.328225 Hartree
Imaginary Frequency: none

16	-4.980988000	-1.628306000	-0.543363000
6	-3.252452000	-1.326453000	-0.447097000
7	-3.121003000	-0.003821000	-0.116505000
7	-4.243416000	0.773701000	-0.017294000
6	-5.297707000	0.062672000	-0.202464000
16	-2.052245000	-2.411938000	-0.748417000
6	0.037298000	-0.679382000	3.023677000
6	1.142347000	0.168113000	2.929098000
6	1.231670000	1.126044000	1.910067000
6	0.187424000	1.170461000	1.007894000
6	-0.911089000	0.317541000	1.076273000
6	-1.006159000	-0.619939000	2.095109000
7	-1.610128000	1.622521000	-0.795223000
6	-1.867656000	0.660578000	-0.000523000
16	-0.082897000	2.281636000	-0.364970000
8	-0.272956000	3.642862000	0.087094000
8	0.834368000	1.994386000	-1.458257000
6	-6.676652000	0.627292000	-0.131417000
1	-0.003886000	-1.410958000	3.823452000
1	1.971094000	0.074835000	3.622282000
1	2.100782000	1.772188000	1.841174000
1	-1.854153000	-1.294361000	2.156409000
1	-6.604613000	1.694127000	0.082990000
1	-7.199462000	0.484221000	-1.080411000
1	-7.253601000	0.137516000	0.657179000
17	4.372646000	-0.193162000	2.000494000
1	3.884278000	-0.430506000	0.244599000
7	3.761326000	-0.424014000	-0.841428000
6	4.935469000	-1.178046000	-1.365108000
1	4.898803000	-1.147352000	-2.458311000
1	5.814740000	-0.623755000	-1.024677000
6	4.995333000	-2.607770000	-0.852410000
1	4.194934000	-3.225219000	-1.267658000
1	4.941486000	-2.622692000	0.239913000
1	5.947096000	-3.049066000	-1.157592000
6	3.846862000	1.033298000	-1.171410000
1	3.037005000	1.511178000	-0.617531000
1	4.792372000	1.382247000	-0.747877000
6	3.726044000	1.345602000	-2.652856000
1	2.791504000	0.960961000	-3.069452000
1	4.568121000	0.956617000	-3.231031000
1	3.706832000	2.430648000	-2.776562000
6	2.429861000	-1.019392000	-1.197210000
1	1.748259000	-0.184266000	-1.378273000
1	2.544705000	-1.563083000	-2.141169000
6	1.870180000	-1.905076000	-0.091659000
1	0.849580000	-2.194628000	-0.356561000
1	1.840929000	-1.359831000	0.854627000
1	2.462891000	-2.807285000	0.066738000

XXV
SCF energy: −2646.857117 Hartree
Free energy correction: 0.330192 Hartree
Imaginary Frequency: none

16	-0.132180000	3.945683000	0.228202000
6	0.863869000	2.519892000	0.547107000
7	0.679724000	1.668342000	-0.547949000
7	-0.217899000	2.073176000	-1.516635000
6	-0.703212000	3.229478000	-1.257640000
16	1.723992000	2.352816000	1.918810000
6	4.615893000	-0.501898000	0.530099000
6	4.547053000	-1.885234000	0.367357000
6	3.391451000	-2.480488000	-0.144281000
6	2.327903000	-1.646042000	-0.419696000
6	2.348503000	-0.270870000	-0.202441000
6	3.525793000	0.321378000	0.239906000
7	0.251984000	-0.454808000	-1.312897000
6	1.053252000	0.311016000	-0.666391000
16	0.843914000	-2.034091000	-1.322616000
8	1.199025000	-2.417683000	-2.678705000
8	-0.109482000	-2.861676000	-0.595812000
6	-1.674989000	3.909351000	-2.163586000
1	5.535015000	-0.050804000	0.888487000
1	5.406162000	-2.501118000	0.611623000
1	3.333663000	-3.546578000	-0.335032000
1	3.602573000	1.392222000	0.366174000
1	-1.879770000	3.255430000	-3.012295000
1	-2.607155000	4.122223000	-1.632954000
1	-1.265954000	4.855608000	-2.527168000
17	-0.177949000	-0.554756000	1.979230000
1	-1.610199000	-1.111527000	0.774199000
7	-2.631857000	-1.274772000	0.468953000
6	-2.961830000	-2.681423000	0.870164000
1	-3.990952000	-2.879647000	0.555726000
1	-2.282197000	-3.306581000	0.287269000
6	-2.780134000	-2.949875000	2.356905000
1	-3.549800000	-2.466550000	2.963675000
1	-1.795178000	-2.614907000	2.691964000
1	-2.860097000	-4.027685000	2.518638000
6	-2.750328000	-1.095275000	-1.013200000
1	-2.215292000	-0.179608000	-1.268727000
1	-2.189060000	-1.919627000	-1.456656000
6	-4.184683000	-1.068493000	-1.521248000
1	-4.751322000	-0.221281000	-1.125564000
1	-4.730050000	-1.987343000	-1.292305000
1	-4.158048000	-0.968574000	-2.608977000
6	-3.384644000	-0.250276000	1.255865000
1	-4.453555000	-0.471286000	1.165412000
1	-3.074006000	-0.389393000	2.292127000
6	-3.041238000	1.169336000	0.827035000
1	-3.499164000	1.868410000	1.530984000
1	-3.402343000	1.409753000	-0.177042000
1	-1.955728000	1.307031000	0.873280000

XXVI
SCF energy: −2646.831332 Hartree
Free energy correction: 0.332296 Hartree
Imaginary Frequency: −93.8398 cm^{-1}

16	0.187187000	3.926973000	0.410238000

6	-0.880194000	2.634804000	-0.151547000
7	-0.433377000	1.464971000	0.447777000
7	0.696765000	1.561914000	1.233064000
6	1.120962000	2.769447000	1.314714000
16	-2.086110000	2.924603000	-1.214127000
6	-4.679462000	-0.431107000	0.305531000
6	-4.666459000	-1.819233000	0.437420000
6	-3.451403000	-2.500768000	0.512334000
6	-2.297719000	-1.746697000	0.418628000
6	-2.282686000	-0.364487000	0.239404000
6	-3.499109000	0.309513000	0.207895000
7	0.027195000	-0.786080000	0.613670000
6	-0.846217000	0.106520000	0.190180000
16	-0.628399000	-2.303446000	0.650932000
8	-0.454624000	-2.881421000	1.979980000
8	-0.108620000	-3.084553000	-0.471846000
6	2.322954000	3.140680000	2.119221000
1	-5.628724000	0.093798000	0.276197000
1	-5.600102000	-2.367821000	0.503202000
1	-3.404507000	-3.574814000	0.658442000
1	-3.541285000	1.382657000	0.096010000
1	2.710427000	2.245677000	2.609404000
1	3.097361000	3.571141000	1.477496000
1	2.065382000	3.879667000	2.882306000
17	-0.595666000	0.055259000	-2.127923000
1	1.612530000	-0.799999000	-0.283608000
7	2.638879000	-1.032075000	-0.273092000
6	2.844995000	-2.239206000	-1.146183000
1	3.905403000	-2.498108000	-1.077674000
1	2.243894000	-3.029627000	-0.691716000
6	2.408757000	-2.032971000	-2.584848000
1	3.075694000	-1.358430000	-3.128034000
1	1.382517000	-1.655706000	-2.629222000
1	2.437954000	-3.003852000	-3.085347000
6	2.865463000	-1.368482000	1.176701000
1	2.503236000	-0.500672000	1.734097000
1	2.192286000	-2.197686000	1.410535000
6	4.306519000	-1.716931000	1.509154000
1	5.002234000	-0.916639000	1.241562000
1	4.631471000	-2.639997000	1.024339000
1	4.380688000	-1.872289000	2.587791000
6	3.418488000	0.181668000	-0.695506000
1	3.666725000	0.722007000	0.222662000
1	4.353663000	-0.174178000	-1.139619000
6	2.659886000	1.108238000	-1.633556000
1	3.291285000	1.977185000	-1.840873000
1	1.727387000	1.452448000	-1.181098000
1	2.389015000	0.636506000	-2.577104000

XXVII
SCF energy: −2646.840349 Hartree
Free energy correction: 0.330641 Hartree
Imaginary Frequency: none

16	-1.354116000	-3.484887000	-0.859933000
6	-1.788807000	-2.147180000	0.207480000
7	-0.873112000	-1.151171000	-0.017477000
7	0.072193000	-1.375384000	-0.981086000
6	-0.048229000	-2.539143000	-1.513125000
16	-3.092509000	-2.256654000	1.214435000
6	-4.116803000	1.979998000	-0.443355000
6	-3.570010000	3.041220000	-1.170755000
6	-2.188189000	3.176844000	-1.285322000
6	-1.404316000	2.212515000	-0.672171000
6	-1.926386000	1.140899000	0.033882000
6	-3.307191000	1.025833000	0.173384000
7	0.424344000	0.813888000	0.338679000
6	-0.813278000	0.248340000	0.589689000
16	0.367291000	2.154650000	-0.575396000
8	0.968879000	1.935031000	-1.900126000
8	0.898390000	3.314480000	0.147070000
6	0.845895000	-3.025344000	-2.607568000
1	-5.194525000	1.900592000	-0.343689000
1	-4.224573000	3.772167000	-1.634206000
1	-1.734417000	4.004529000	-1.820457000
1	-3.735301000	0.221891000	0.759522000
1	1.418739000	-2.182395000	-2.997425000
1	1.529653000	-3.797402000	-2.241773000
1	0.256613000	-3.453538000	-3.422002000
17	-1.038472000	0.037676000	2.412525000
1	1.852718000	0.138266000	0.348228000
7	2.938763000	-0.038435000	0.301698000
6	3.512331000	1.268181000	0.761556000
1	4.602737000	1.182548000	0.731797000
1	3.196168000	2.016088000	0.026703000
6	3.024971000	1.662460000	2.147428000
1	3.389880000	0.979744000	2.920628000
1	1.933570000	1.704790000	2.176761000
1	3.405303000	2.661118000	2.373051000
6	3.221911000	-0.299186000	-1.144828000
1	2.763731000	-1.267615000	-1.357670000
1	2.667039000	0.457287000	-1.708754000
6	4.703818000	-0.301497000	-1.487296000
1	5.276424000	-0.972200000	-0.837922000
1	5.138943000	0.698217000	-1.426553000
1	4.827624000	-0.649271000	-2.515549000
6	3.302245000	-1.192065000	1.186110000
1	3.628369000	-2.012611000	0.541801000
1	4.158637000	-0.898055000	1.800015000
6	2.123027000	-1.628208000	2.042849000
1	2.421185000	-2.461259000	2.684015000
1	1.288214000	-1.959185000	1.416421000
1	1.757239000	-0.814324000	2.672427000

XXVIII
SCF energy: −2646.820739 Hartree
Free energy correction: 0.332414 Hartree
Imaginary Frequency: −175.1690 cm^{-1}

16	-1.824367000	-2.759216000	-1.284367000
6	-1.786661000	-2.011156000	0.333531000
7	-0.718701000	-1.197331000	0.400478000
7	0.048351000	-1.134992000	-0.717250000
6	-0.386036000	-1.869419000	-1.689588000
16	-2.937282000	-2.332985000	1.499392000
6	-4.058923000	1.535285000	-0.399753000
6	-3.591758000	2.258982000	-1.500679000
6	-2.224632000	2.477690000	-1.686028000
6	-1.369958000	1.924480000	-0.749255000
6	-1.819727000	1.188859000	0.339950000
6	-3.181145000	0.996355000	0.542974000
7	0.521118000	1.165401000	0.791414000
6	-0.656128000	0.695682000	1.142724000
16	0.395158000	2.107082000	-0.582397000
8	1.149244000	1.500338000	-1.685985000
8	0.738721000	3.486032000	-0.258049000
6	0.271462000	-1.918662000	-3.032006000
1	-5.126433000	1.389455000	-0.272165000
1	-4.300105000	2.669321000	-2.212864000
1	-1.847544000	3.063610000	-2.517563000
1	-3.536546000	0.427407000	1.394858000
1	0.892745000	-1.027976000	-3.148056000
1	0.885907000	-2.818100000	-3.146504000
1	-0.479859000	-1.926351000	-3.825224000
17	-0.909700000	0.345989000	2.832665000
1	1.946126000	-0.091389000	0.170440000
7	2.956871000	-0.339546000	0.113202000
6	3.728610000	0.933624000	0.337342000
1	4.774273000	0.700746000	0.122681000
1	3.358599000	1.629721000	-0.421163000

6	3.597372000	1.527920000	1.730372000
1	4.035992000	0.882289000	2.495816000
1	2.555812000	1.739667000	1.975554000
1	4.149741000	2.470766000	1.737203000
6	3.164189000	-0.851640000	-1.284932000
1	2.483409000	-1.698999000	-1.379895000
1	2.804864000	-0.059517000	-1.945007000
6	4.597623000	-1.257016000	-1.590102000
1	4.995300000	-1.963219000	-0.855401000
1	5.272481000	-0.401596000	-1.656220000
1	4.610023000	-1.754908000	-2.562519000
6	3.210798000	-1.400595000	1.151582000
1	3.181553000	-2.361777000	0.633078000
1	4.227011000	-1.252602000	1.528634000
6	2.167700000	-1.382152000	2.258321000
1	2.407371000	-2.165585000	2.981634000
1	1.170003000	-1.580464000	1.854486000
1	2.134126000	-0.425509000	2.781485000

XXIX

SCF energy: −2646.833940 Hartree
Free energy correction: 0.331598 Hartree
Imaginary Frequency: none

16	-1.948717000	-2.069928000	-1.461471000
6	-1.608123000	-2.103042000	0.295008000
7	-0.416133000	-1.565413000	0.530047000
7	0.230493000	-1.119345000	-0.583754000
6	-0.422156000	-1.288168000	-1.697660000
16	-2.726885000	-2.680404000	1.416413000
6	-3.988980000	0.797584000	-0.529828000
6	-3.526105000	1.376358000	-1.713591000
6	-2.212316000	1.847085000	-1.826529000
6	-1.403315000	1.693221000	-0.718116000
6	-1.852908000	1.121127000	0.468472000
6	-3.160051000	0.665922000	0.586575000
7	0.370893000	1.631785000	1.151394000
6	-0.759869000	1.115505000	1.465180000
16	0.261502000	2.270670000	-0.425007000
8	1.273325000	1.610483000	-1.249333000
8	0.286317000	3.719941000	-0.352515000
6	0.091903000	-0.856701000	-3.034753000
1	-5.006771000	0.426511000	-0.477870000
1	-4.195522000	1.463125000	-2.563101000
1	-1.849077000	2.311778000	-2.737152000
1	-3.503120000	0.186996000	1.497315000
1	0.834489000	-0.068308000	-2.894292000
1	0.540395000	-1.695991000	-3.578054000
1	-0.721208000	-0.461454000	-3.650357000
17	-1.035066000	0.492506000	3.029401000
1	2.000961000	-0.255455000	-0.105379000
7	3.009970000	-0.526698000	-0.046721000
6	3.887555000	0.691227000	-0.101664000
1	4.911105000	0.324610000	-0.211054000
1	3.594561000	1.220141000	-1.009802000
6	3.790701000	1.614153000	1.099993000
1	4.202524000	1.156609000	2.002822000
1	2.762846000	1.924270000	1.294531000
1	4.382280000	2.506270000	0.880178000
6	3.195026000	-1.355673000	-1.289232000
1	2.411116000	-2.113776000	-1.248420000
1	2.969210000	-0.690367000	-2.127047000
6	4.573653000	-1.981032000	-1.430047000
1	4.839183000	-2.589695000	-0.561863000
1	5.360249000	-1.242475000	-1.596404000
1	4.558406000	-2.642158000	-2.299543000
6	3.181661000	-1.349505000	1.206279000
1	2.983215000	-2.386351000	0.923560000
1	4.234484000	-1.268683000	1.494840000
6	2.229294000	-0.949624000	2.322958000
1	2.443076000	-1.578052000	3.191943000
1	1.195172000	-1.135999000	2.016503000
1	2.334637000	0.094129000	2.619616000
